# Prenatal PM_2.5_ exposure impairs spatial learning and memory in male mice offspring: from transcriptional regulation to neuronal morphogenesis

**DOI:** 10.1186/s12989-023-00520-2

**Published:** 2023-04-20

**Authors:** Yanwen Hou, Wei Yan, Lin Guo, Guangke Li, Nan Sang

**Affiliations:** 1 College of Environment and Resource, Research Center of Environment and Health, Shanxi University, Taiyuan, Shanxi 030006 PR China; 2 Xuzhou Engineering Research Center of Medical Genetics and Transformation, Key Laboratory of Genetic Foundation and Clinical Application, Department of Genetics, Xuzhou Medical University, Xuzhou, Jiangsu 221004 PR China

**Keywords:** Fine particulate matter (PM_2.5_) prenatal exposure, Spatial learning and memory, mRNA expression profiles, Neuronal morphogenesis, Homeobox A5 (Hoxa5)

## Abstract

**Background:**

As one of the environmental risk factors for human health, atmospheric fine particulate matter (PM_2.5_) contributes to cognitive deterioration in addition to respiratory and cardiovascular injuries. Recently, increasing evidence implicates that PM_2.5_ inhalation can affect neurological functions in offspring, but the sex-specific outcomes and the underlying biological processes are largely unknown.

**Objectives:**

To observe the influence of prenatal PM_2.5_ exposure on cognitive performance in offspring, to elucidate the neuronal morphological alterations and possible transcriptional regulation based on mRNA-sequencing (mRNA-Seq) data after birth, and to determine the key components of PM_2.5_ contributing to the adverse effects.

**Methods:**

Pregnant C57BL/6J mice were exposed to sterile saline or PM_2.5_ suspension. Morris water maze test was used to assess the cognitive function in weanling offspring. Microscopic observation was applied to detect neuronal morphogenesis in vivo and in vitro. The cortex tissues from male offspring were collected on postnatal days (PNDs) 1, 7, and 21 for mRNA-Seq analysis. The organic and inorganic components of PM_2.5_ were separated to assess their contributions using primary cultured neurons.

**Results:**

Prenatal PM_2.5_ exposure impaired spatial learning and memory in weanling male mice, but not female mice. The sex-specific outcomes were associated with mRNA expression profiles of the cortex during postnatal critical windows, and the annotations in Gene Ontology (GO) of differentially expressed genes (DEGs) revealed that the exposure persistently disrupted the expression of genes involved in neuronal features in male offspring. Consistently, axonal growth impairment and dendritic complexity reduction were observed. Importantly, Homeobox A5 (Hoxa5), a critical transcription factor regulating all of the neuronal morphogenesis-associated hub genes on PNDs 1, 7, and 21, significantly decreased in the cortex of male offspring following PM_2.5_ exposure. In addition, both inorganic and organic components were harmful to axonal and dendritic growth, with organic components exhibiting stronger inhibition than inorganic ones.

**Conclusion:**

Prenatal PM_2.5_ exposure affected spatial learning and memory in male mice by disrupting Hoxa5-mediated neuronal morphogenesis, and the organic components, including polycyclic aromatic hydrocarbons (PAHs), posed more adverse effects than the inorganic components.

**Supplementary Information:**

The online version contains supplementary material available at 10.1186/s12989-023-00520-2.

## Background

Air pollution has been considered the largest environmental health risk in the world that causes over 4,200,000 deaths annually according to the World Health Organization (WHO) [1]. Particulate matter less than 2.5 μm in diameter (PM_2.5_) is one of the most hazardous air pollutants [2–6]. Above 90% of people in the world breathe PM_2.5_ which exceeds WHO air quality guidelines in daily life [7]. In China, PM_2.5_ is a major air pollutant in 337 cities, and the days with PM_2.5_ account for 77.7% of the polluted or heavily-polluted days [8]. Recently, epidemiological studies have shown that air pollution is one of the important factors for developmental neurotoxicity and may contribute to neurodevelopmental disorders [9–12]. For instance, a cohort study of 320 children in Mexico City shows that maternal exposure to PM_2.5_ during pregnancy reduces neurocognitive performance in children 9–10 years of age, and the middle-late gestation may be the most sensitive period [13].

Brain development is a complex and continuous process that undergoes substantial structural changes, including neurogenesis, neuronal maturation, and synaptic plasticity [14]. Large populations of neurons are connected by axons and dendrites, and they communicate with each other through synaptic transmission and form neuronal circuits [15], which constitute the biochemical machinery and mediate key neuronal properties such as learning and memory [16, 17]. An in vivo experiment reveals that PM_2.5_ treatment during pregnancy may disrupt normal cortical development in mice offspring [18]. However, there is a tremendous lack of studies on the impacts of early-life exposure to PM_2.5_ on the morphological development of neurons at postnatal windows.

The pathogenesis of cognitive disorders involves a great number of biological processes, which can be traced back to genetic alterations in different life stages [19, 20]. Although many studies have shown that prenatal PM_2.5_ exposure causes cognitive impairment in mice offspring, these studies are conducted only at a single developmental window instead of a series of windows critical for neurodevelopment [21, 22]. Additionally, these studies show that differentially expressed genes (DEGs) are involved in some signal transduction pathways, such as oxidative stress, inflammatory response, and the CREB/BDNF and HMGB1-NLRP3 signaling pathways [21, 22]. However, it is still unclear whether and how these signaling pathways disturb neurodevelopmental processes. Therefore, it is necessary to investigate transcriptional changes directly induced by prenatal PM_2.5_ exposure in offspring’s critical neurodevelopmental windows and to elucidate the underlying regulatory mechanisms.

In this study, we established an animal model by exposing pregnant C57BL/6 mice to PM_2.5_ to explore the following issues: (1) whether prenatal PM_2.5_ exposure adversely affected spatial learning and memory in offspring in a sex-dependent manner, (2) which biological processes involved at different developmental time points, (3) what were the related molecular targets and potential regulatory mechanisms, and (4) which components of PM_2.5_, organic or inorganic, played crucial roles in neuronal morphogenesis.

## Results

### Physical and chemical characteristics of PM_2.5_ samples

PM_2.5_ were highly diverse in their morphology, consisting of agglomerated soot aggregates, regular and irregular mineral particles, spherical particles, etc. (Fig. S1a-c). Dynamic light scattering (DLS) analyses showed that the hydrodynamic diameter of particles in suspension was 600.1 ± 30.25 nm, with a zeta potential of -33.03 ± 0.61 mV and a polydispersity index (PDI) of 0.785 ± 0.044 (Fig. S1d). There were 15 polycyclic aromatic hydrocarbons (PAHs) and 31 elements in PM_2.5_, and their concentrations are listed in Table S1. The top three abundant PAHs included Benzo(b)fluoranthene (BbFA, 16.70 ng/m^3^), Fluoranthene (FA, 14.58 ng/m^3^), and Chrysene (CHR, 12.20 ng/m^3^). The top four abundant heavy metals included Zn (323.87 ng/m^3^), Cu (194.62 ng/m^3^), Pb (97.94 ng/m^3^), and Mn (93.76 ng/m^3^).

### Prenatal PM_2.5_ exposure impaired spatial learning and memory in weanling male mice

No significant differences were observed in brain-to-body ratio, body length, and tail length between the vehicle group and the prenatal PM_2.5_-exposed group (Fig. S2b-d). However, prenatal PM_2.5_ exposure reduced the body weight of male offspring on postnatal days (PNDs) 1 and 7, but not female offspring (Fig. S2a), which was consistent with our previous result [23].

**Fig. 1:**
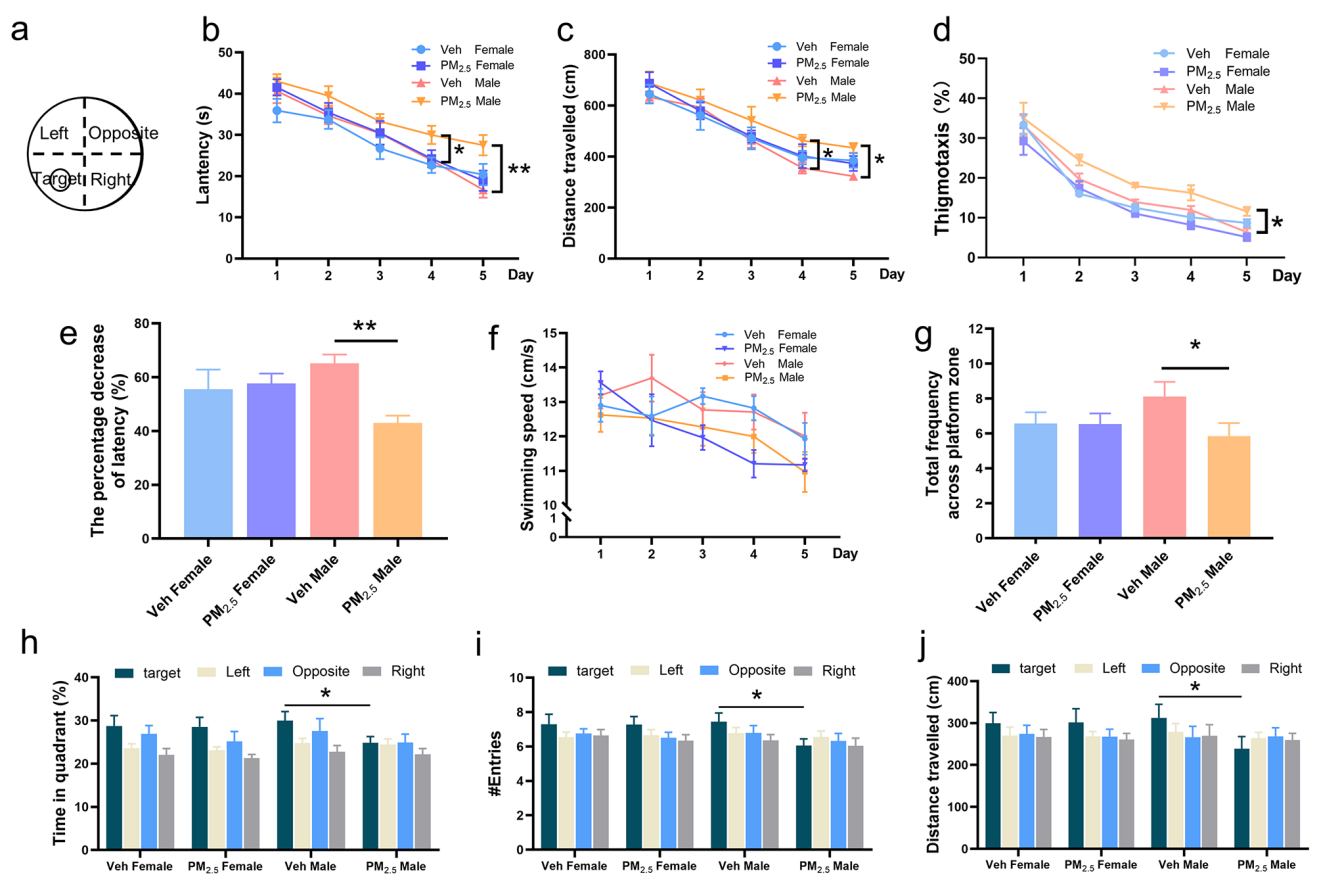
Effects of prenatal PM_2.5_ exposure on spatial learning and memory in weanling offspring. (a) Morris water maze diagram. Learning performance was assessed by (b) escape latency, (c) distance travelled, (d) thigmotaxis, (e) percentage decrease of latency, and (f) swimming speed during the first 5 days. Memory function was assessed by (g) the total frequency across platform zone in the target quadrant, (h) the time in quadrant (%), (i) the number of times crossing the quadrant, and (j) distance travelled in four quadrants during the memory probe test. n = 13–27 from 10 litters

To evaluate whether prenatal PM_2.5_ exposure induced adverse effects on cognitive function, the offspring were subjected to Morris water maze (MWM) at approximately 22 days of age (range from PND 21 to 23), and a simplified diagram of the MWM was shown in Fig. 1a. We investigated the escape latency (s), travel distance (cm), thigmotaxis (%), and swimming speed (cm/s) during the training phase from day 1 to day 5, and found that prenatal PM_2.5_ exposure increased the escape latency and distance of swimming on the fourth and fifth training days in male offspring (Fig. 1b and c), as well as the thigmotaxis on the fifth training day 5 (Fig. 1d). Likewise, the latency from day 1 to day 5 had obvious less improvement in the PM_2.5_-exposed male offspring (Fig. 1e). However, there were no significant differences in swimming speed (Fig. 1f).

A no-platform probe test was conducted on the 6th day. Prenatal PM_2.5_ exposure dramatically decreased the number of times through the platform zone in male offspring, but not female offspring (Fig. 1g). Similarly, the exposure significantly decreased the percentage of time in the target quadrant (Fig. 1h), times of entering the platform zone (Fig. 1i), and the distance travelling in the target quadrant (Fig. 1j) in male offspring. These results demonstrated that prenatal PM_2.5_ exposure caused male-specific spatial learning and memory impairment in offspring, suggesting that males might be more vulnerable than females in terms of neurocognitive outcomes.

### Prenatal PM_2.5_ exposure altered mRNA transcriptome associated with neuronal development in male offspring

**Fig. 2:**
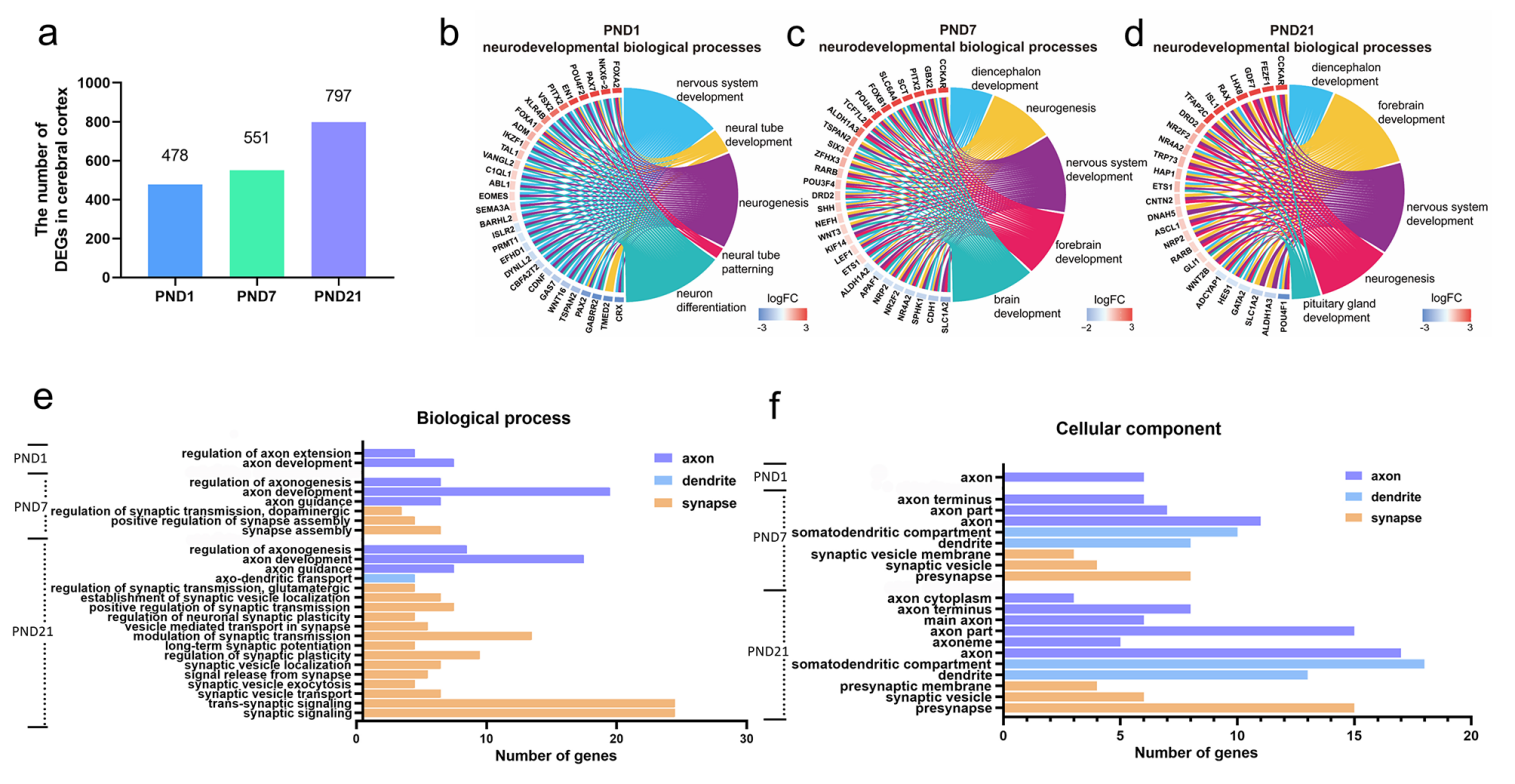
Analysis of mRNA expression profiles in the cortex of male offspring following prenatal PM_2.5_ exposure. (a) Bar graphs showing the number of cortical DEGs at different windows. Chord plot depicting the relationship between DEGs and neurodevelopment-related biological process GO terms on PNDs 1 (b), 7 (c) and 21 (d). Neurodevelopment-related GO terms: biological process (e) and cellular component (f) in male offspring. n = 3 from 3 litters

To elucidate transcriptional regulation of male-specific spatial learning and memory impairment following prenatal PM_2.5_ exposure, we performed mRNA-sequencing (mRNA-seq) on cortical tissues of male offspring at postnatal developmental windows. In detail, prenatal PM_2.5_ exposure caused differential expression of 478, 551, and 797 genes on PNDs 1, 7, and 21, respectively (Fig. 2a, fold change ≥ 1.5; *p* < 0.05), indicating that the number of DEGs gradually increased during postnatal development. To gain insights into the relationship between DEGs and possible neurodevelopmental alterations, we performed gene ontology (GO) analyses using the database for annotation, visualization and integrated discovery (DAVID). The results showed that these DEGs were functionally annotated into GO categories related to neurodevelopmental processes (*p* < 0.05, Excel Table S1), and the top 5 significantly enriched biological processes included nervous system development (GO:0007399), neural tube development (GO:0021915), neurogenesis (GO:0022008), neuron differentiation (GO:0030182), and forebrain development (GO:0030900) (Fig. 2b-d).

The neuron is the smallest unit in the nervous system [24]. The neuronal development includes axon formation, dendrite outgrowth, and synaptogenesis, which are the cornerstone of cognitive function later in life [25]. During postnatal development, the biological processes associated with axonal and dendritic development included axon development (PNDs 1, 7, and 21), regulation of axon extension (PND 1), regulation of axonogenesis (PNDs 7 and 21), axon guidance (PNDs 7 and 21), and axo-dendritic transport (PND 21). For synaptic development, the significantly enriched GO terms of biological processes were highly associated with synapse assembly and signal transmission at the late stages of neurodevelopment (PNDs 7 and 21), such as synapse assembly (PND 7), regulation of synaptic transmission, dopaminergic (PND 7), modulation of synaptic transmission (PND 21), regulation of synaptic plasticity (PND 21), synaptic vesicle transport (PND 21), etc. (Fig. 2e). In addition, the DEGs were significantly enriched in cellular components at each developmental window. On PND 1, the DEGs were only enriched in the axon-related category. On PNDs 7 and 21, the DEGs were enriched in the axon, axon terminus, dendrite, somatodendritic compartment, presynapse, synaptic vesicle, and synaptic vesicle membrane (Fig. 2f). The GO terms of biological processes enriched by neuronal development-associated DEGs on PNDs 1, 7, and 21 are listed in Excel Table S2. The results suggested that these abnormally expressed neuronal genes might regulate the development of axons, dendrites and synapses, and they played an important role in PM_2.5_ exposure-induced cognitive deficits in male offspring.

### Prenatal PM_2.5_ exposure impaired neuronal morphogenesis in male offspring

**Fig. 3:**
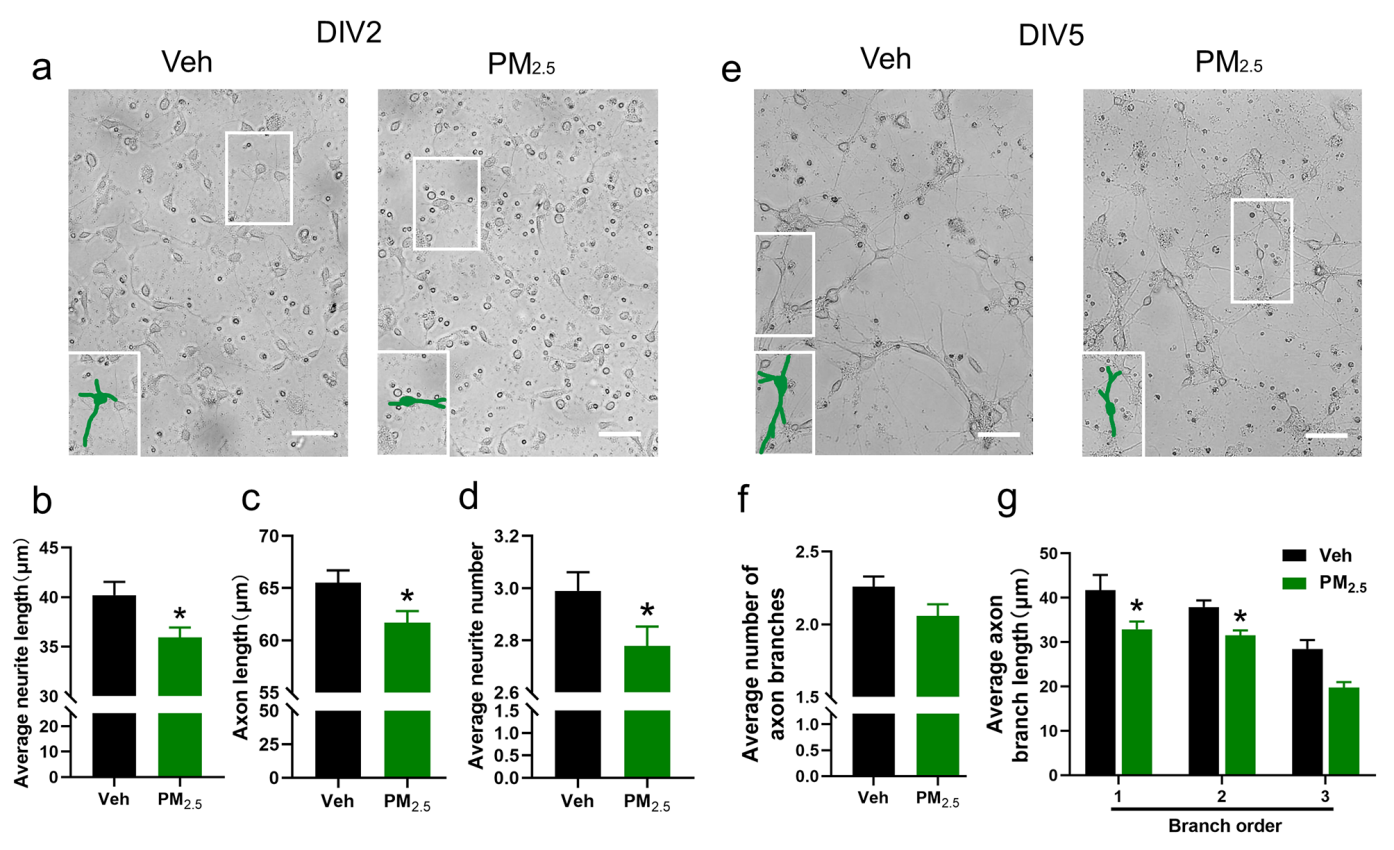
Effects of prenatal PM_2.5_ exposure on morphological development of axons in male offspring. (a) Representative images of primary cortical neurons on DIV2. Bar = 50 μm; (b) Average neurite length; (c) Axon length; and (d) Average neurite number. n = 96 (Veh) and 95 (PM_2.5_) neurons from 3 to 5 litters. (e) Representative images of primary cortical neurons on DIV5. Bar = 50 μm; (f) Average number of axon branches; (g) Average axon branch length at different branch orders. n = 50 (Veh) and 52 (PM_2.5_) neurons from 3 to 5 litters

Since the morphological development of neurons (including axons, dendrites, and synapses) is critical to neuronal function [26], we further observed the changes of neuronal morphology in male offspring following prenatal PM_2.5_ exposure. Firstly, we observed axonal morphology on the second day in vitro (DIV2) and the fifth day in vitro (DIV5) using primary cultured cortical neurons isolated from newborn male offspring, and representative images are shown in Fig. 3a and e. Specifically, prenatal PM_2.5_ exposure significantly decreased the average neurite length, axon length, and average neurite number (Fig. 3b-d) on DIV2. There was no significant difference in the average number of axon branches on DIV5, but the exposure remarkably decreased the average axon branch length in the first and second branches (Fig. 3f and g).

**Fig. 4:**
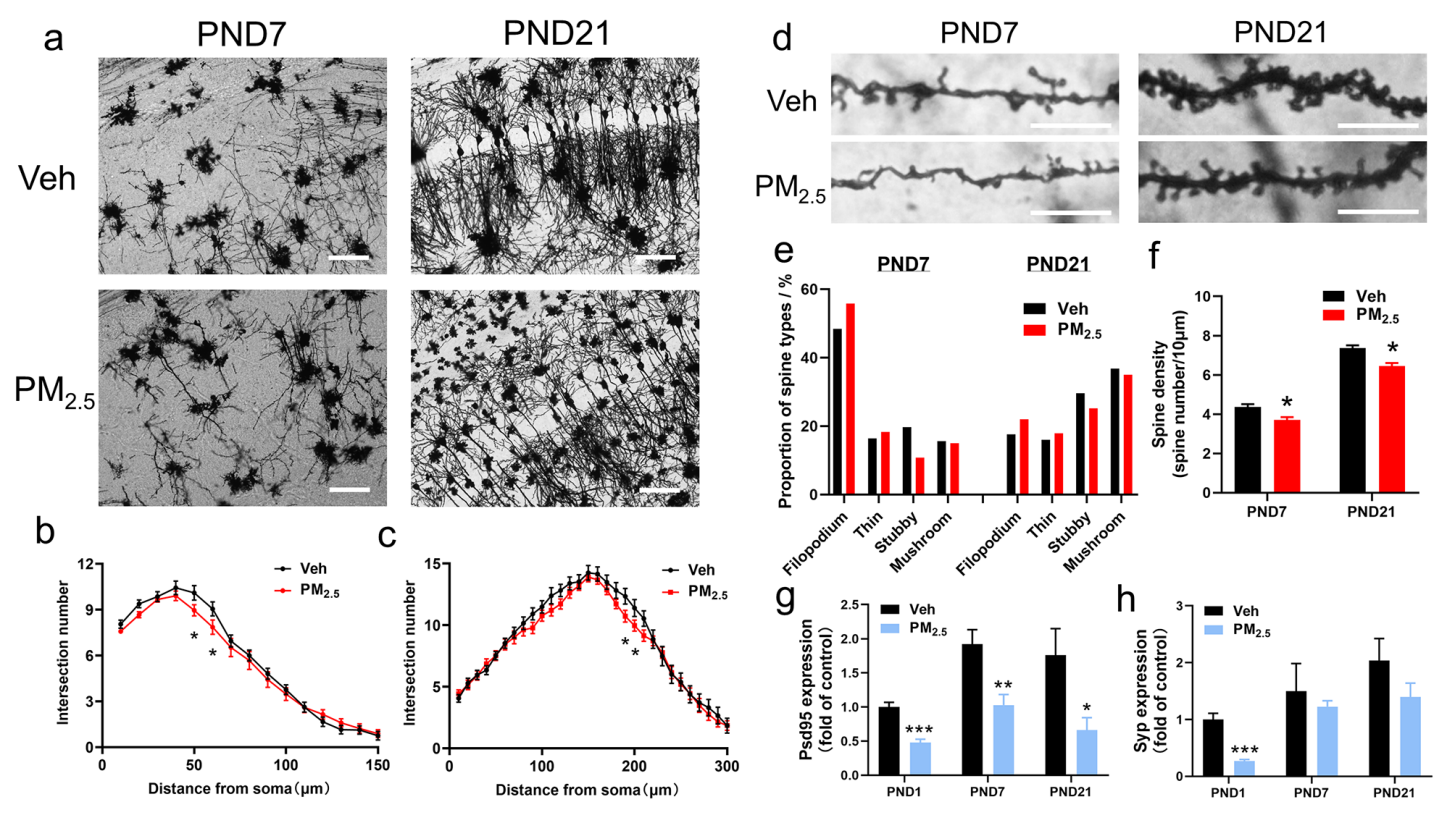
Effects of prenatal PM_2.5_ exposure on morphological development of dendrites and mRNA expression of synaptic markers in male offspring. (a) Representative images of Golgi-Cox-stained dendrites on PNDs 7 and 21. Bar = 200 μm; (b) Sholl analyses of dendritic complexity on PND 7 (n = 21 neurons from 3 mice of different litters); (c) Sholl analyses of dendritic complexity on PND 21 (n = 21 neurons from 3 mice of different litters). (d) Representative images of Golgi-Cox stained dendritic spines on PNDs 7 and 21. Bar = 10 μm. (e) The percentage of each spine morphological category (filopodium, thin, stubby, and mushroom) on PNDs 7 and 21 (n = 15 neurons from 3 mice of different litters). (f) Quantification of dendritic spine density was calculated as the number of spines per 10-µm dendrite length (n = 17–20 neurons from 3 mice of different litters). (g) mRNA expression of Psd95 on PNDs 1, 7, and 21. (h) mRNA expression of Syp on PNDs 1, 7, and 21. (n = 4–6 from 4 to 6 litters)

Secondly, we observed the dendritic morphology in the male offspring on PNDs 7 and 21 using Golgi-Cox staining and measured the number of dendritic branches, the proportion of spine types, and spine density. As shown in Fig. 4a-c, the number of dendritic branches was considerably reduced at 50–60 μm and 190–200 μm from the cell body in male offspring on PNDs 7 and 21. Dendritic spines are classified into four types by shape: filopodium, thin, stubby, and mushroom [27]. Among them, filopodium and thin spines are regarded as immature dendritic spines, and stubby and mushroom spines are regarded as mature dendritic spines. Here, we quantified the number of different types of dendritic spines on PNDs 7 and 21. The results showed that prenatal PM_2.5_ exposure reduced the proportions of stubby spines, increased the proportions of filopodium spines, and considerably reduced the density of dendritic spines (Fig. 4d-f). These results demonstrated that prenatal PM_2.5_ exposure impaired the morphological development of axons and significantly decreased dendritic complexity and maturation in male offspring.

Dendritic spines form the postsynaptic component of the most excitatory synapses in the brain and play a critical role in synaptic transmission and plasticity, which were thought to underlie learning and memory [28]. Therefore, we further examined the expression of synaptic elements that were closely related to the formation and maturation of synapse-postsynaptic density protein 95 (Psd95) and synaptophysin (Syp). As shown in Fig. 4g and h, prenatal PM_2.5_ exposure remarkably reduced the expression of Psd95 on PNDs 1, 7, and 21 and decreased the expression of Syp on PND 1, suggesting axon and dendrite-coupled deterioration of synaptic function in male offspring.

### Hoxa5 regulated abnormal neuronal morphogenesis in male offspring

**Fig. 5:**
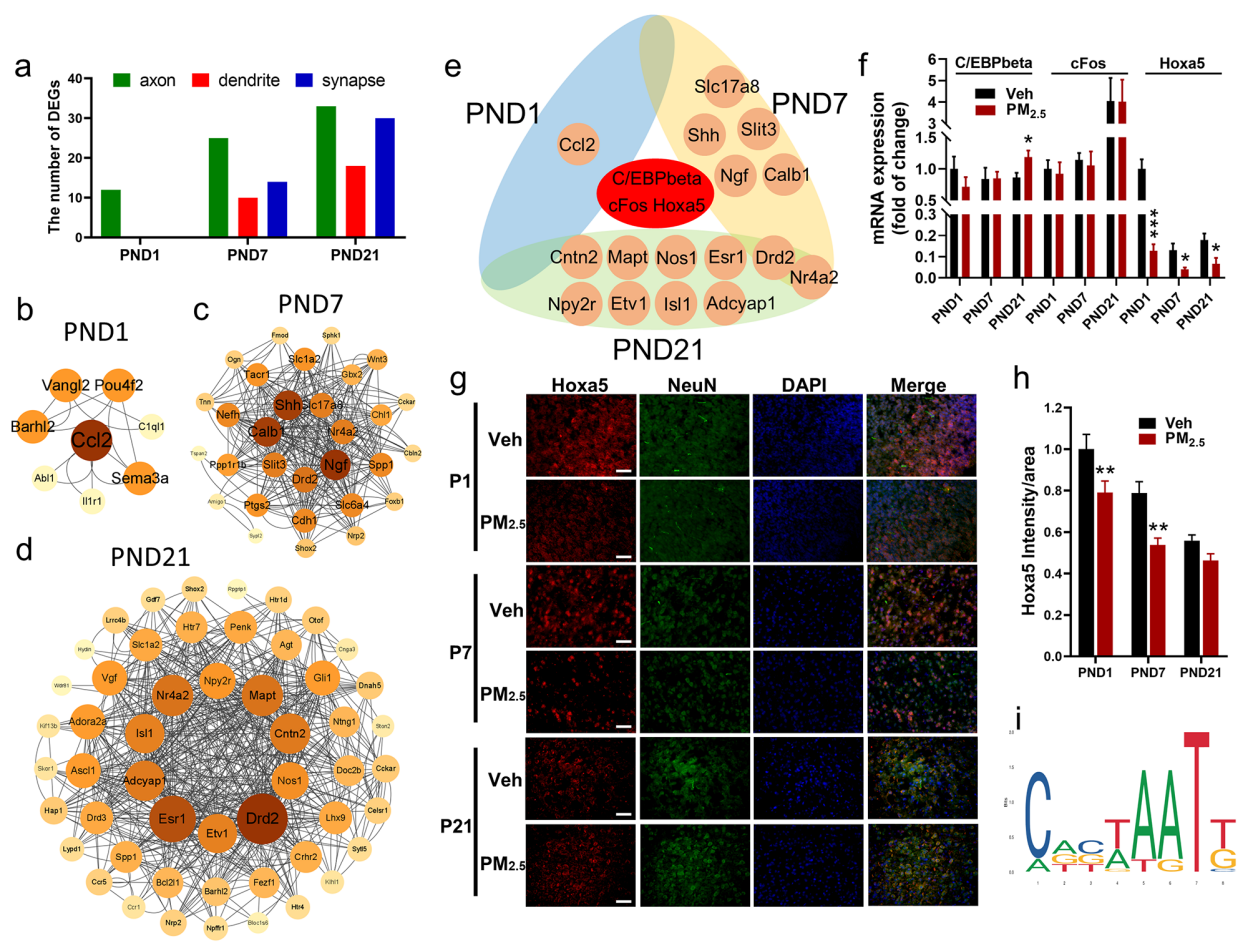
Transcriptional regulation of neuronal morphological development-associated DEGs in male offspring following prenatal PM_2.5_ exposure. (a) The numbers of DEGs associated with the morphological development of axons, dendrites, and synapses at different windows, with genes selected based on FDR < 0.05. (b-d) PPI of DEGs associated with axons, dendrites, and synapses on PNDs 1, 7 and 21, with inner circles representing the hub genes. (e) The upstream transcriptional factors of the hub genes on PNDs 1, 7 and 21. (f) Relative mRNA levels of C/EBPbeta, cFos and Hoxa5 on PNDs 1, 7 and 21 (n = 4–6 from 4–6 litters). (g) Immunofluorescence staining of HOXA5 (red fluorescence) on PNDs 1, 7 and 21, with neurons stained with NeuN (green fluorescence) and nuclei fluorescence) and nuclei stained with DAPI (blue fluorescence) (n = 3 from 3 litters), Bar = 50 μm. (h) Quantification of the immunofluorescence intensity of Hoxa5 on PNDs 1, 7 and 21. (i) A diagram showing the binding sites of Hoxa5 with the hub genes on PNDs 1, 7 and 21 using the JASPAR database

To elucidate the possible transcriptional mechanisms underlying prenatal PM_2.5_ exposure-caused DEGs-driving abnormal neuronal morphogenesis and spatial learning and memory impairment in male offspring, we determined the upstream transcription factors (TFs) which could regulate the expression of DEGs involved in neuronal development. Firstly, we identified the DEGs modulating morphological plasticity of axons, dendrites, and synapses on PNDs 1, 7, and 21, and the results are shown in Fig. 5a. On PND 1, we identified 12 genes that were only associated with axonal development, but not dendritic and synaptic growth. On PNDs 7 and 21, however, we identified 33 and 54 DEGs, respectively, and they were associated with the development of axons, dendrites, and synapses. Here, we verified the expression of the top 30 downregulated DEGs by qPCR, and found that the exposure decreased the expression of these DEGs to varying extents at different postnatal developmental windows (Fig. S3), which was consistent with the mRNA-seq result (Table S2).

Next, we mapped the 12, 33, and 54 DEGs on PNDs 1, 7, and 21 to the STRING database to construct protein-protein interaction (PPI) networks, respectively, and to identify the hub genes at each developmental window following the exposure. With the required confidence (combined score) > 0.15 (low confidence), there were a total of 8, 30, and 52 nodes and 28, 548, and 1092 interactions that were identified on PNDs 1, 7, and 21, respectively. As shown in Fig. 5b-d, based on the degree of each node, the top 20% of the genes in the PPI network, such as Ccl2 on PND1, Slc17a8, Nr4a2, Ngf, Drd2, Slit3, Calb1 and Shh on PND7, Drd2, Etv1, Esr1, Adcyap1, Isl1, Nr4a2, Npy2r, Mapt, Cntn2 and Nos1 on PND21, were considered as the hub genes. Furthermore, we used the PROMO database to explore the TFs that might regulate the expression of the hub genes, and obtained 3 TFs (Hoxa5, cFos, and C/EBPbeta) that regulated all of the hub genes (Fig. 5e). All upstream TFs predicted for each hub gene are shown in Table S3. We next tested mRNA expression of Hoxa5, cFos and C/EBPbeta and found that only Hoxa5 significantly decreased on PNDs 1, 7, and 21, but cFos and C/EBPbeta showed no changes (Fig. 5f). Consistently, prenatal PM_2.5_ exposure dramatically decreased the protein level of Hoxa5 in neurons (Fig. 5g and h). Importantly, the binding site of Hoxa5 to the hub gene promoter region was further predicted using the JASPAR CORE database, and the results are shown in Fig. 5i and Table S4. These findings implicated that Hoxa5 regulated neuronal morphogenesis-coded genes and contributed to impaired spatial learning and memory in male offspring.

### Effects of PM_2.5_ components on neuronal morphogenesis and transcriptional regulation

**Fig. 6:**
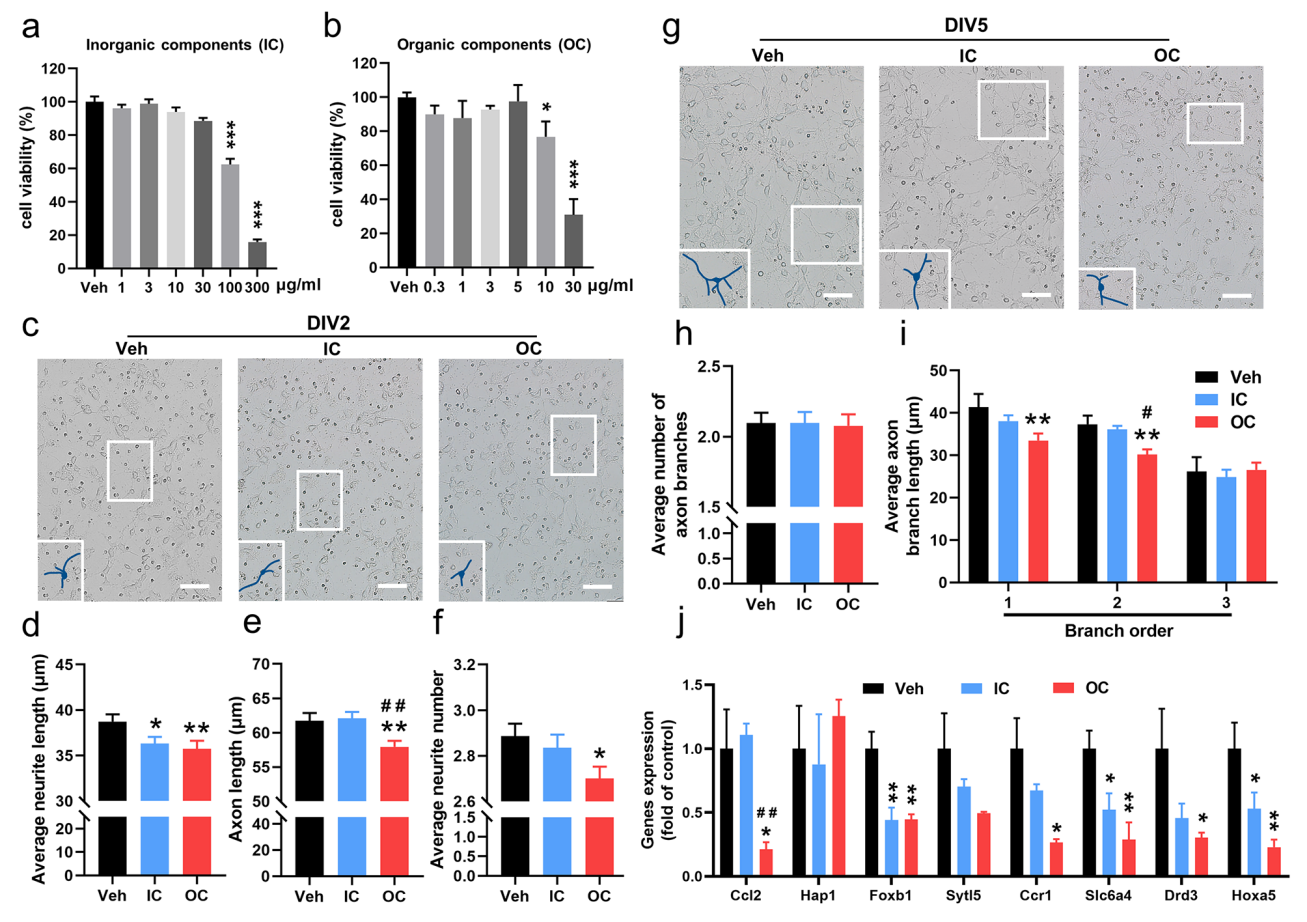
Effects of inorganic components (IC) and organic components (OC) of PM_2.5_ on neuronal morphology and gene expression. (a and b) The cytotoxicity of IC and OC to primary cultured cortical neurons. (c) Representative images of primary cortical neurons on DIV2. Bar = 50 μm; (d-f) The average neurite length, axon length, and average neurite number. n = 97 (Veh), 97 (IC) and 97 (OC) neurons from 3 to 5 litters. (g) Representative images of primary cortical neurons on DIV5. Bar = 50 μm; (h and i) The average number of axon branches and the average length of axon branches at different branches. n = 52 (Veh), 52 (IC) and 52 (OC) neurons from 3 to 5 litters. (j) mRNA expression of genes related to neuronal morphological development (n = 3 from 3 litters). Data are presented as mean ± SEM from three independent experiments. *p < 0.05, **p < 0.01 for comparisons between the PM_2.5_ exposure group and the vehicle group; ^#^p < 0.05, ^##^p < 0.01 for comparisons between the IC group and the OC group

To identify the critical components of PM_2.5_ contributing to the above neuronal morphogenesis and transcriptional regulation, we separated the inorganic components (IC) and organic components (OC, mainly including PAHs) and used them to treat primary cultured cortical neurons isolated from male neonatal mice. Based on in vitro cytotoxicity assays on DIV1, no cytotoxic effects were observed below 30 µg/ml for IC and 5 µg/ml for OC, so 30 and 5 µg/ml were considered as the maximum non-lethal concentration for IC and OC, respectively (Fig. 6a and b). By treating the neurons with IC and OC at the non-lethal concentrations on DIV1 and observing axonal morphology on DIV2 and DIV5, we found that OC significantly decreased the average neurite length, axon length, and average neurite number on DIV2, whereas IC only significantly decreased the average neurite length on DIV2 (Fig. 6c-f). In addition, there was no significant difference in the average number of axon branches after both IC and OC treatment, but the average axon branch length in the first and second branches remarkably declined in the OC group on DIV5 (Fig. 6g-i). It was worth noting that the axon length on DIV2 and the second branch length on DIV5 showed more decrease in the OC group than that in the IC group.

To confirm the morphological changes described above, we further examined mRNA expressions of 7 down-regulated DEGs and Hoxa5 on DIV5. These 7 down-regulated DEGs were associated with neuronal development, with fold changes greater than 2 in RNA-Seq analysis. As shown in Fig. 6j, OC significantly decreased the mRNA levels of Ccl2, Foxb1, Ccr1, Slc6a4, Drd3, and Hoxa5, and IC just reduced that of Foxb1, Slc6a4 and Hoxa5. Importantly, OC (including PAHs) exhibited stronger effects than IC.

## Discussion

As reported, prenatal exposure to atomized nano-PM [29], ambient ultrafine particle [30], PM_2.5_ [31–33], and traffic-related black carbon [34] was associated with male-specific neurodevelopmental impairments, such as decreased neuronal differentiation, viability and density, impaired neuronal morphological features, as well as cognitive, emotional and behavioral deficits [29–33]. However, it was also reported that a 10-µg/m^3^ increase in PM_2.5_ concentration during pregnancy was significantly correlated with a higher risk of developmental stunting for children, particularly within the field of problem-solving for girls [35]. Zhao et al. demonstrated that gestational PM_2.5_ exposure induced cognitive and emotional disorders in female mice offspring [36].

In the present study, we found that prenatal PM_2.5_ exposure did deteriorate spatial learning and memory in male mice offspring but not female offspring. Psychiatric and clinical literature has reinforced that sex differences in the brain play a crucial role in the sex bias of incidence, severity, and progression of numerous neurodevelopmental disorders [37]. Importantly, males are more susceptible than females to the adverse consequences of in-utero air pollution exposure, and they exhibit more apparent neurodevelopmental disorders in early life and subsequent impairment of cognition and behavior in childhood [38]. The reason may be that males have more microglia during gestational and early postnatal periods [39], which would amplify the inflammatory response in the fetal brain derived from their mothers who are exposed to PM_2.5_ during early pregnancy [40]. Besides learning and memory dysfunction, our unpublished data also indicated that the exposure induced male-specific anxiety and autism as well as the gradually increased number of differentially expressed genes in the cortex as the embryo developed, but not the females, further implicating that prenatal PM_2.5_ exposure resulted in sex-different neurodevelopmental outcomes and male offspring were more susceptible than females. Our finding was in line with a recent epidemiological study conducted by Lertxundi et al. in Spain, which reported that boys were more susceptible to PM_2.5_ exposure during pregnancy than girls, especially in domains related to memory and cognition [41].

During cortical development in newborns, neuronal differentiation begins when they migrate in the proper position. One of the neurites rapidly extends and grows into an axon, while the others develop into dendrites. When a neuron’s axon makes contact with a potential dendrite, it undergoes dramatic structural changes to form synaptic structures that transmit information. Numerous neurons form complex neural circuits via presynaptic and postsynaptic connections [42–45]. In our study, the DEGs in male offspring following prenatal exposure to PM_2.5_ were mainly enriched in biological processes related to neuronal development, such as neurogenesis, neuronal differentiation, and neuron projection development, as well as morphological features of neurons, such as axon extension, axonogenesis, axo-dendritic transport, somatodendritic compartment, and synapse assembly. The formation of neuronal networks depends upon the structural development of neurons, especially axonal and dendritic morphogenesis, which play an essential role in the establishment of functional neural networks and neurocognitive function [46, 47]. These reports support our finding that prenatal PM_2.5_ exposure contributed to the male-specific spatial learning and memory impairment by altering the mRNA expressions associated with axonal and dendritic development.

The formation of axons and dendrites is important in the establishment of functional neural circuits [48], which are thought to be associated with behavior and functional dysfunction [49]. Axons are very thin nerve fibers that carry nerve impulses away from one neuron to another. Axonal outgrowth is essential for wiring the central nervous system during development [50]. Numerous studies found that axonal injury is a critical initiating event in a variety of central nervous system diseases, which is implicated in the neuropathology of depression, cognitive dysfunction, metabolic encephalopathies, and multiple sclerosis [51, 52]. The present study indicated that prenatal PM_2.5_ exposure decreased axon length, axon branch and neurite number, which might contribute to cognitive impairment in male offspring.

Dendrite branching and morphology facilitate allow individual neurons to receive inputs, process information, and carry out specialized brain functions and cognitive behaviors, such as social networking, learning, and memory [53]. A growing number of reports have indicated that alterations in dendrite morphology, including changes in dendrite branching patterns, retraction or loss of dendrite branches, contribute to various neurological, neurodevelopmental, and cognitive disorders, such as autism, anxiety, depression, schizophrenia, mental retardation, Parkinson’s disease, and Alzheimer’s disease [53]. Especially, the structural plasticity of dendritic spines is the basis of memory and cognition of the brain [54, 55]. In this study, we demonstrated that prenatal PM_2.5_ exposure significantly reduced the number of dendritic branches at 50–60 μm and 190–200 μm, disturbed the proportions of spine types, and decreased the spine density in the brain of male offspring, suggesting that the exposure impaired dendritic morphological development at postnatal periods and contributed to cognitive disorders.

In addition to the results of male offspring, we also observed the morphological development of axons and dendrites and determined mRNA expressions on PNDs 1, 7, and 21 in female offspring following prenatal PM_2.5_ exposure. The results showed that there were no significant differences in morphological features, such as average neurite length, axon length, and average neurite number on DIV2 (Fig. S4a-d); axon branches number and average axon branch length on DIV5 (Fig. S4e-g); the number of dendritic branches, the proportion of spine types, and spine density (Fig. S5a-f). Also, except for Slc6a4 and Drd3 on PND 7, there were no significant differences in the gene expression in the cortex of female offspring, including 5 down-regulated DEGs associated with neuronal development with fold changes greater than 2 in transcriptome sequencing analyses of male offspring, and synaptic markers Psd95 and Syp on PNDs 1, 7, and 21 (Fig. S5g and h, Fig. S6). By combining the results of postnatal neuronal morphology and cortical mRNA expressions in male and female offspring on postnatal days, we found that prenatal PM_2.5_ exposure induced sex-specific neurodevelopmental outcomes, especially in the expression of genes associated with neuronal features and the morphogenesis in male offspring, but the exposure showed limited effects on females. Therefore, this might be the critical reason for the sex-specific spatial learning and memory deterioration following prenatal PM_2.5_ exposure.

To elucidate the possible regulatory mechanism, we found different DEGs during postnatal neurodevelopment, but these hub genes shared a common upstream transcription factor - Hoxa5. Hoxa5 is a member of the HOX gene family and contributes to the continuous development of the embryonic and fetal central nervous system from neurulation to the establishment of functional neuronal networks, including normal tangential migration of pontine neurons, proper axonal projection and arborization, survival of neurons, establishment and plasticity of neural circuits [56]. Here, Hoxa5 regulated almost all of the DEGs associated with the axonal and dendritic morphogenesis, and the prenatal PM_2.5_ exposure dramatically decreased its level in the cortex of male offspring, but not females (Fig. 5f, Fig. S6).

PM_2.5_ is a mixture consisting of organic compounds and inorganic metals [57, 58]. In the present study, we found that both organic and inorganic extracts from PM_2.5_ showed inhibitory effects on neuronal growth as well as transcriptional regulation, and significantly decreased Hoxa5 levels. Interestingly, the organic extracts caused stronger effects than inorganic extracts, possibly due to the presence of PAHs in coal-burning areas.

## Conclusion

In the current study, prenatal PM_2.5_ exposure caused spatial learning and memory impairment in male mice offspring but not female offspring. The impairment was associated with altered gene expression involved in axonal and dendritic morphogenesis. Importantly, Hoxa5 regulated all of the hub genes, most of them were significantly decreased in male offspring following prenatal PM_2.5_ exposure and played a key role in the male-specific neuronal morphogenesis and cognitive dysfunction. Furthermore, both inorganic and organic components of PM_2.5_ caused adverse effects on neuronal morphogenesis, and the organic components (including PAHs) exhibited stronger inhibition than the inorganic components. Our work provides insights into cognitive dysfunction caused by atmospheric PM_2.5_ pollution.

## Methods

### PM_2.5_ collection and physicochemical characterization

PM_2.5_ samples were collected on the rooftop of a building on the campus of Shanxi University in Taiyuan, Shanxi province (Longitude 112°21–34’, Latitude 37°47–48’) using a middle-volume air sampler (TH-150CIII, China) loaded with quartz fiber filters (F90 mm, Sweden) from November 1, 2018 to March 31, 2019. There were no large obstacles and no large pollution sources near the sampling point. Samples were stored at -20 ℃ before use. The extraction of PM_2.5_ was conducted according to our previous reports [59], and the obtained PM_2.5_ powder was stored at -80 ℃ until use.

The morphology of PM_2.5_ was observed using transmission electron microscopy (JEM-F20, Japan). The hydrodynamic diameter of PM_2.5_ in suspension was measured by a Malvern Zetasizer Nano ZS (Malvern, U.K.). The concentrations of polycyclic aromatic hydrocarbons (PAHs) and heavy metals in PM_2.5_ samples were determined using gas chromatography-mass spectrometry (GC-MS) and inductively coupled plasma mass spectrometry (ICP-MS), respectively.

### Extraction of inorganic and organic components from PM_2.5_

Inorganic and organic extracts were prepared according to the extraction protocol by Xu et al. [60]. For inorganic components, the quartz filters containing PM_2.5_ samples were immersed in ultrapure water, ultrasonicated, mixed well by vortexing, and extracted at 200 rpm for 16 h. The aqueous extracts were filtered through a 0.45 mm filter, freeze-dried, and diluted to a 10 mg/mL stock solution using sterile ultrapure water. For organic components, the quartz filters with PM_2.5_ were extracted using organic solvents (methanol, acetone, n-hexane, and acetonitrile), and the extracts were collected and evaporated in a rotary evaporator to a constant weight. Then, the organic extracts were separated using reverse-phase high-performance liquid chromatography (RP-HPLC). The chromatographic collection times were calibrated using PAHs mixed standards and sulfonamide antibiotics mixed standards, and the fractions containing PAHs were collected and dissolved in dimethyl sulfoxide (DMSO) to a 10 mg/mL stock solution for subsequent cytotoxicity analysis.

### Animals and prenatal PM_2.5_ exposure

Six-eight-week-old mice (C57BL/6J, 90 male and 180 female) were purchased from the Beijing Vital River Laboratory Animal Technology (Beijing, China) and reared under similar conditions described in our previous study [20]. Animal experiments were approved by the Committee of Scientific Research at Shanxi University (Approval No.: SXULL2019025). The detailed mating scheme was provided in the Supplemental Material (Text S1). Pregnant animals were exposed to either PM_2.5_ (3 mg/kg every two days) or sterile saline via oropharyngeal aspiration, as described in our previous study [61]. Mice offspring were euthanized with isoflurane on PNDs 1, 7, and 21, and cortical tissues were collected for further analysis, with at least 8 pups/group (1–2 pups per dam) randomly selected from 6 dams at each time point. In addition, behavioral tests were performed starting on PND 21 with 13–27 pups/group, and these pups were randomly selected from 10 dams.

### Behavioral tests

The Morris water maze (MWM) was performed to test spatial reference learning and memory in offspring on PNDs 21–23. The detailed procedures of training and testing were described in our previous study [20].

### Transcriptome sequencing analyses

Three male offspring were randomly selected from three dams in each group for mRNA-seq analyses, which were performed by Shanghai Biotechnology Corporation (Shanghai, China). The detailed protocol for mRNA-seq was provided in our previous study [20]. Gene Ontology (GO) enrichment analysis for all DEGs or neuronal development-related DEGs was conducted using DAVID (https://david.ncifcrf.gov/summary.jsp).

### Hub genes screening and TF prediction

PPI network analysis was performed at different time points through the STRING database (https://string-db.org/), and the top 20% of genes were defined as the hub genes based on the degree of each node. Then, the hub genes were imported into the PROMO database (http://alggen.lsi.upc.es/cgi-bin/promo_v3/promo/promoinit.cgi?dirDB=TF_8.3) with the maximum matrix dissimilarity rate of 0 to predict their possible TFs.

### Real-time quantitative polymerase chain reaction (RT-qPCR)

Total RNA was extracted from the cerebral cortex of offspring on PNDs 1, 7, and 21 using TRIZOL Reagent (Invitrogen, USA). RNA was quantified using a Nanodrop 2000c spectrophotometer (Thermo Fisher Scientific, USA), and cDNA was synthesized using a Reverse Transcription Kit (Takara, China). The gene-specific primers were obtained from PrimerBank (https://pga.mgh.harvard.edu/primerbank/) (Table S5). Finally, the expression of genes was determined using RT-qPCR (TaKaRa, China), and GAPDH was used as an internal control.

### Cultivation of primary cortical neurons and observation of axonal morphology

Briefly, cortical tissues were dissected from the pups of different dams within 24 h after birth, digested with trypsin, and mechanically triturated in Neurobasal/B27 medium (Invitrogen, USA). Then, neurons were centrifuged, re-suspended in Neurobasal/B27 medium, and cultured in an incubator. Half of the Neurobasal medium was changed every three days. The detailed processes were described in our previous study [62]. Axonal morphology was observed on DIV2 and DIV5 using an inverted microscope (IX73, Olympus, Japan) and measured using Image J.

### Golgi-Cox staining

Brains were dissected and fixed with 4% paraformaldehyde, sliced and immersed in a Golgi-Cox staining solution (G1069, Servicebio, China) for 2 weeks, followed by fixation with 80% glacial acetic acid and dehydration with 30% sucrose. Brain slides were observed using an IX73 inverted microscope (Olympus Corporation, Japan). About 20 neurons with intact dendritic trees were measured from 3 offspring in each group, and dendritic complexity was determined using Sholl analysis. The number of intersections within concentric circles at 10-µm intervals from the soma was counted [63]. The neuronal morphology was quantified using ImageJ.

### Immunofluorescence staining

The brains were fixed with 4% paraformaldehyde and dehydrated in 10%, 20% and 30% sucrose until they sank to the bottom. Twenty-µm-thick frozen sections were blocked with 3% BSA for 1 h and incubated with anti-Hoxa5 (DF4123, Affinity Biosciences) and anti-NeuN antibody (MAB377, Merck) at 4 ℃ overnight. The next day, the slides were washed 3 times using PBS and incubated with secondary antibodies for 1 h. Nuclei were stained with 4’,6-diamidino-2-phenylindole (DAPI, KGA215-10, KeyGEN BioTECH). The slides were observed using a fluorescence microscope (IX73, Olympus, Japan), and the fluorescence intensity of Hoxa5 was quantified using ImageJ.

### In vitro cytotoxicity assay

The in vitro cytotoxicity of IC and OC from PM_2.5_ on primary cultured cortical neurons was detected using the cell counting kit-8 (CCK-8, minibio, China). The detailed protocol was provided in Supplemental Material (Text S2).

### Statistical analysis

All data are presented as mean ± SEM. Differences among different groups were evaluated using one-way analyses of variance (ANOVA), followed by the Fisher’s least significant difference (LSD) test. When *p* < 0.05, differences were considered statistically significant (*p < 0.05. **p < 0.01). Data were analyzed using Origin 2019b and figures were made using GraphPad Prism 8.0.2.

## Electronic supplementary material

Below is the link to the electronic supplementary material.

Excel table S1. Full list of neurodevelopment-related GO terms of biological process based on cortical DEGs in male offspring on PNDs 1, 7 and 21 following prenatal PM_2.5_ exposure. Excel table S2. Full list of GO terms of biological process enriched by neuronal development-associated DEGs on PNDs 1, 7 and 21 in male offspring following prenatal PM_2.5_ exposure.

Additional file 1: Fig. S1. Physical and chemical characteristics of PM_2.5_ samples. Fig. S2. Effects of prenatal PM_2.5_ exposure on birth outcome in offspring. Fig. S3. The heatmap for validating the top 30 down-regulated DEGs. Fig. S4. Effects of prenatal PM_2.5_ exposure on morphological development of axons in female offspring. Fig. S5. Effects of prenatal PM_2.5_ exposure on morphological development of dendrite and mRNA expression of synaptic markers in female offspring. Fig. S6. Effects of prenatal PM_2.5_ exposure on the expression of genes related to neuronal morphological development in female offspring. Table S1. The contents of elements and polycyclic aromatic hydrocarbons in PM_2.5_ samples. Table S2. The top 30 downregulated DEGs associated with the development of axons, dendrites and synapses in mRNA-seq. Table S3. Correlation analyses between hub genes and their TFs in the cortex of male offspring on PNDs 1, 7 and 21 following prenatal PM_2.5_ exposure. Table S4. Prediction of binding sites between Hoxa5 and hub genes in the cortex of male offspring on PNDs 1, 7 and 21 following prenatal PM_2.5_ exposure. Table S5. Primers used in this study. Text S1. Mating scheme of mice. Text S2. The protocol for in vitro cytotoxicity assay.

## Data Availability

All data and materials are included in the main body of the manuscript or in the Additional files. The datasets generated during the current study are available from the corresponding author on reasonable request.

## Abbreviations

mRNA-Seq: mRNA sequencing
SAL: Sterile saline
Veh: vehicle
MWM: Morris water maze
PND: Postnatal days
DEGs: Differentially expressed genes
GO: Gene Ontology
Hoxa5: Homeobox A5
PM_2.5_: Fine particulate matter
WHO: World Health Organization
DLS: Dynamic light scattering
PDI: Polydispersity index
BbFA: Benzo(b)fluoranthene
FA: Fluoranthene
CHR: Chrysene
DIV2: The second day in vitro
DIV5: The fifth day in vitro
Psd95: Postsynaptic density protein 95
Syp: Synaptophysin
TFs: Transcription factors
PPI: Protein-protein interaction
IC: Inorganic components
OC: Organic components
MNLC: Maximum non-lethal concentration
ASD: Autism spectrum disorder
ADHD: Attention-deficit hyperactivity disorder
PAHs: Polycyclic aromatic hydrocarbons
GC-MS: Gas chromatography - mass spectrometry
ICP-MS: Inductively coupled plasma mass spectrometry
NGS: Next generation sequencing
FPKM: Reads per kilobase per million reads
RT-qPCR: Real-time quantitative polymerase chain reaction
TEM: Transmission electron microscopy
ANOVA: One-way analyses of variance
LSD: Least significant difference
RP-HPLC: Reverse phase high performance liquid chromatography
DMSO: Dimethyl sulfoxide
