# Supplementary material for: Prenatal PM2.5 exposure impairs spatial learning and memory in male mice offspring: from transcriptional regulation to neuronal morphogenesis
Source: Part Fibre Toxicol. 2023 Apr 20;20:13. doi: 10.1186/s12989-023-00520-2 (PMC10116824; doi:10.1186/s12989-023-00520-2)
Supplement: Supplementary file 2 — Additional file 1: Fig. S1. Physical and chemical characteristics of PM2.5 samples. Fig. S2. Effects of prenatal PM2.5 exposure on birth outcome in offspring. Fig. S3. The heatmap for validating the top 30 down-regulated DEGs. Fig. S4. Effects of prenatal PM2.5 exposure on morphological development of axons in female offspring. Fig. S5. Effects of prenatal PM2.5 exposure on morphological development of dendrite and mRNA expression of synaptic markers in female offspring. Fig. S6. Effects of prenatal PM2.5 exposure on the expression of genes related to neuronal morphological development in female offspring. Table S1. The contents of elements and polycyclic aromatic hydrocarbons in PM2.5 samples. Table S2. The top 30 downregulated DEGs associated with the development of axons, dendrites and synapses in mRNA-seq. Table S3. Correlation analyses between hub genes and their TFs in the cortex of male offspring on PNDs 1, 7 and 21 following prenatal PM2.5 exposure. Table S4. Prediction of binding sites between Hoxa5 and hub genes in the cortex of male offspring on PNDs 1, 7 and 21 following prenatal PM2.5 exposure. Table S5. Primers used in this study. Text S1. Mating scheme of mice. Text S2. The protocol for in vitro cytotoxicity assay. [file 12989_2023_520_MOESM2_ESM.docx]

Supplementary Information for

Prenatal PM_2.5_ Exposure Impairs Spatial Learning and Memory in Male Mice Offspring: from Transcriptional Regulation to Neuronal Morphogenesis

Yanwen Hou^1^ *^a^*, Wei Yan^1^ *^b^*, Lin Guo *^a^*, Guangke Li* *^a^*, Nan Sang* *^a^*

1. *These authors are co-first authors of the article.*

*a. College of Environment and Resource, Research Center of Environment and Health,* *Shanxi University,* *Taiyuan, Shanxi 030006, PR China.*

*b. Xuzhou Engineering Research Center of Medical Genetics and Transformation, Key Laboratory of Genetic Foundation and Clinical Application, Department of Genetics, Xuzhou Medical University, Xuzhou, Jiangsu 221004, PR China.*

**This PDF file includes:**

Figures S1 to S6

Tables S1 to S5

Texts S1 to S2

**Other supplementary materials for this manuscript include the following:**

Excel tables S1 and S2


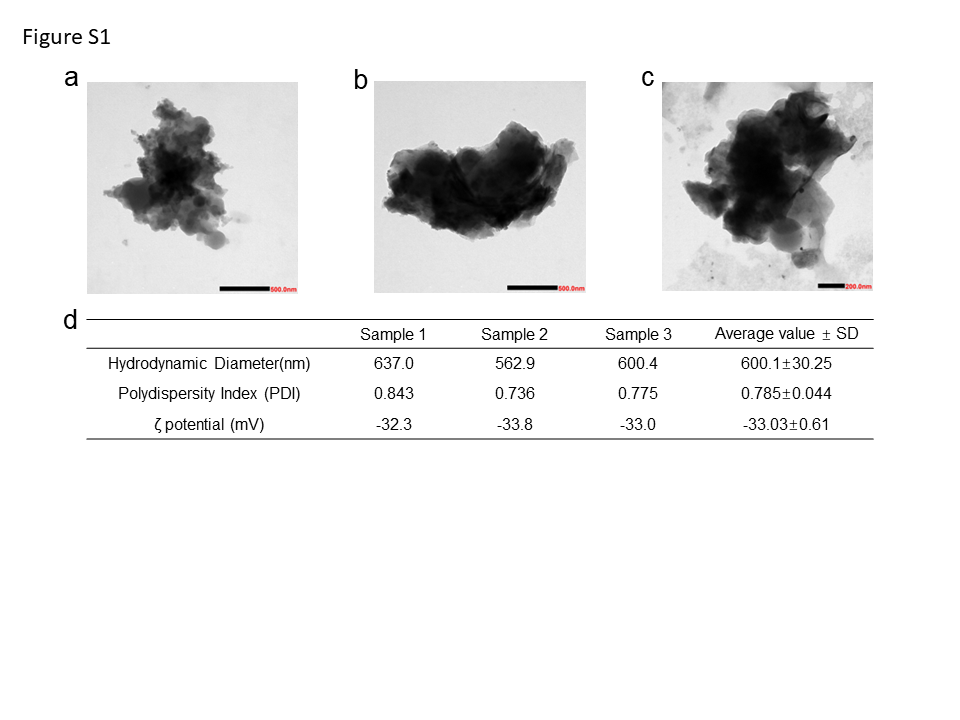


Fig. S1. Physical and chemical characteristics of PM_2.5_ samples. (a-c) Representative transmission electron microscopy (TEM) image. (d) Hydrodynamic diameter, zeta potential and polydispersity index.


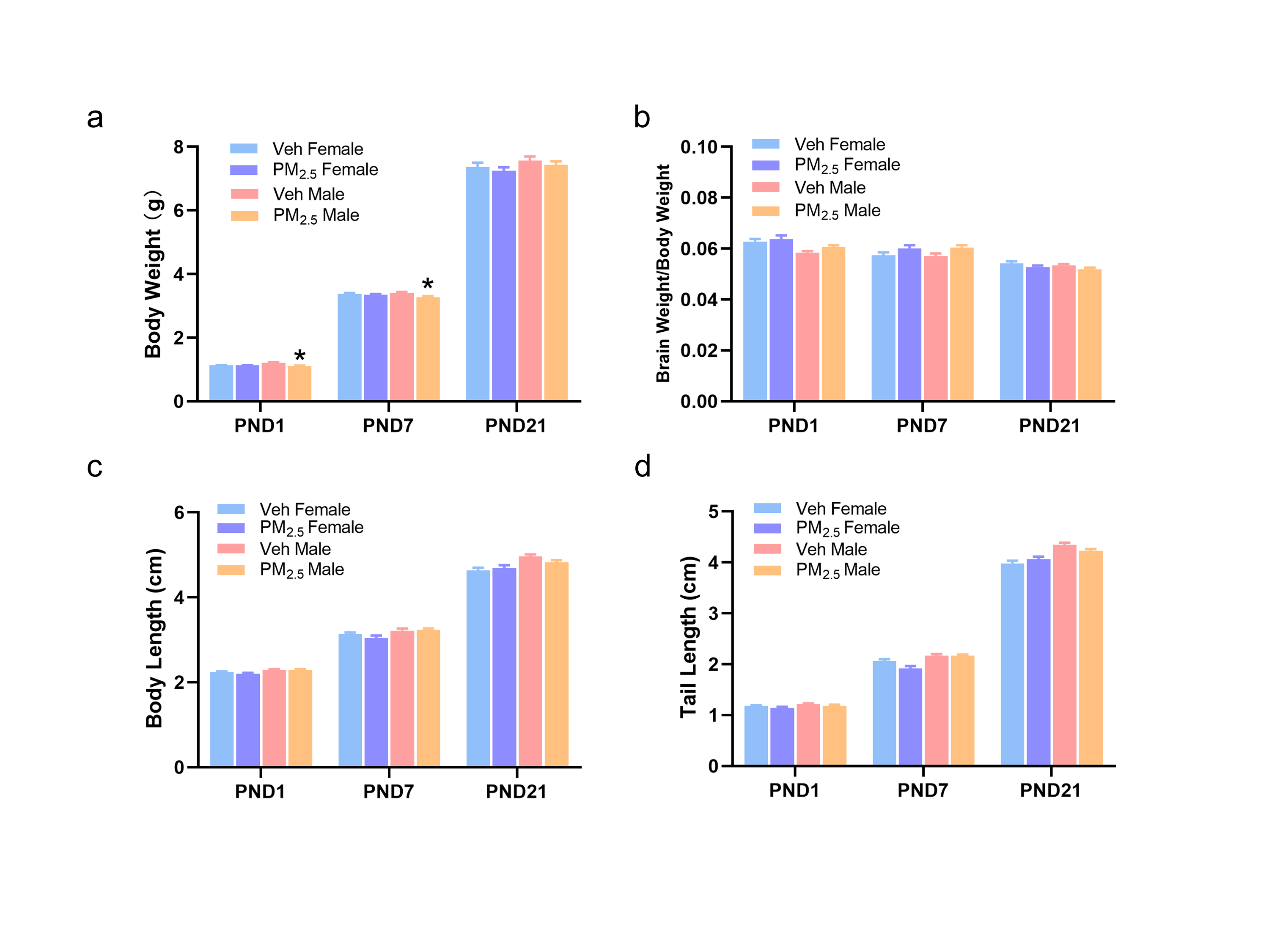


Fig. S2. Effects of prenatal PM_2.5_ exposure on birth outcome in offspring. (a) Body weight (n=21-82), (b) Ratio of viscera to body weight (n=15). (c-d) Body length and tail length (n=16-32). The values represented the mean ± SEM. **p* < 0.05. Abbreviations: Veh, prenatal vehicle control offspring; PM_2.5_, prenatal PM_2.5_-exposed offspring.


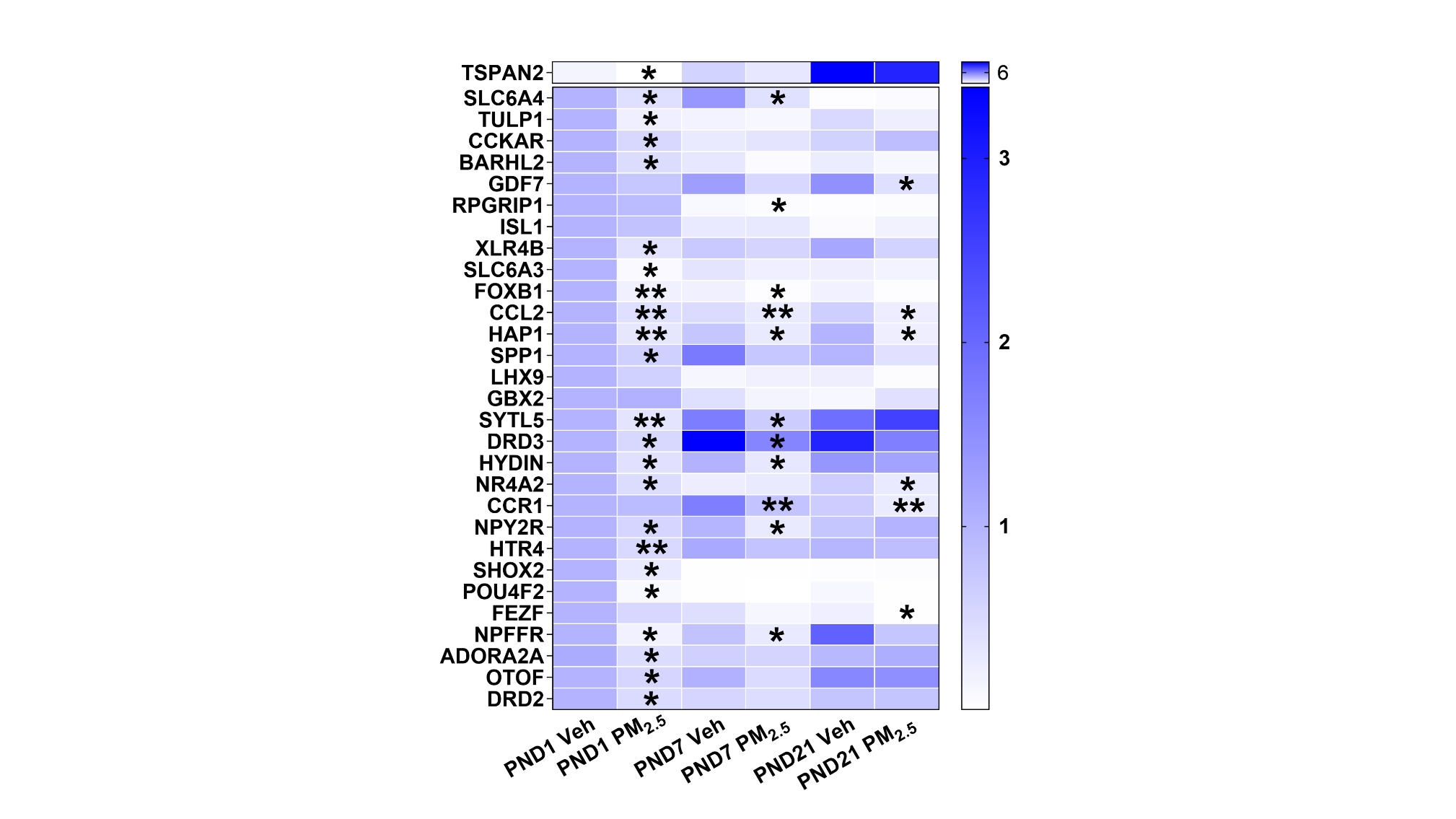


Fig. S3. The heatmap for validating the top 30 down-regulated DEGs (n = 3-6 from 3 to 6 litters). *p<0. 05. **p<0. 01. Abbreviations: Veh, sterile saline-exposed offspring; PM_2.5_, PM_2.5_ suspension-exposed offspring; PND, postnatal day. The genes expression levels in the PND1 vehicle group were assigned a value of 1.0. The depth of color represents the level of gene expression, and the darker the color, the higher the expression value.


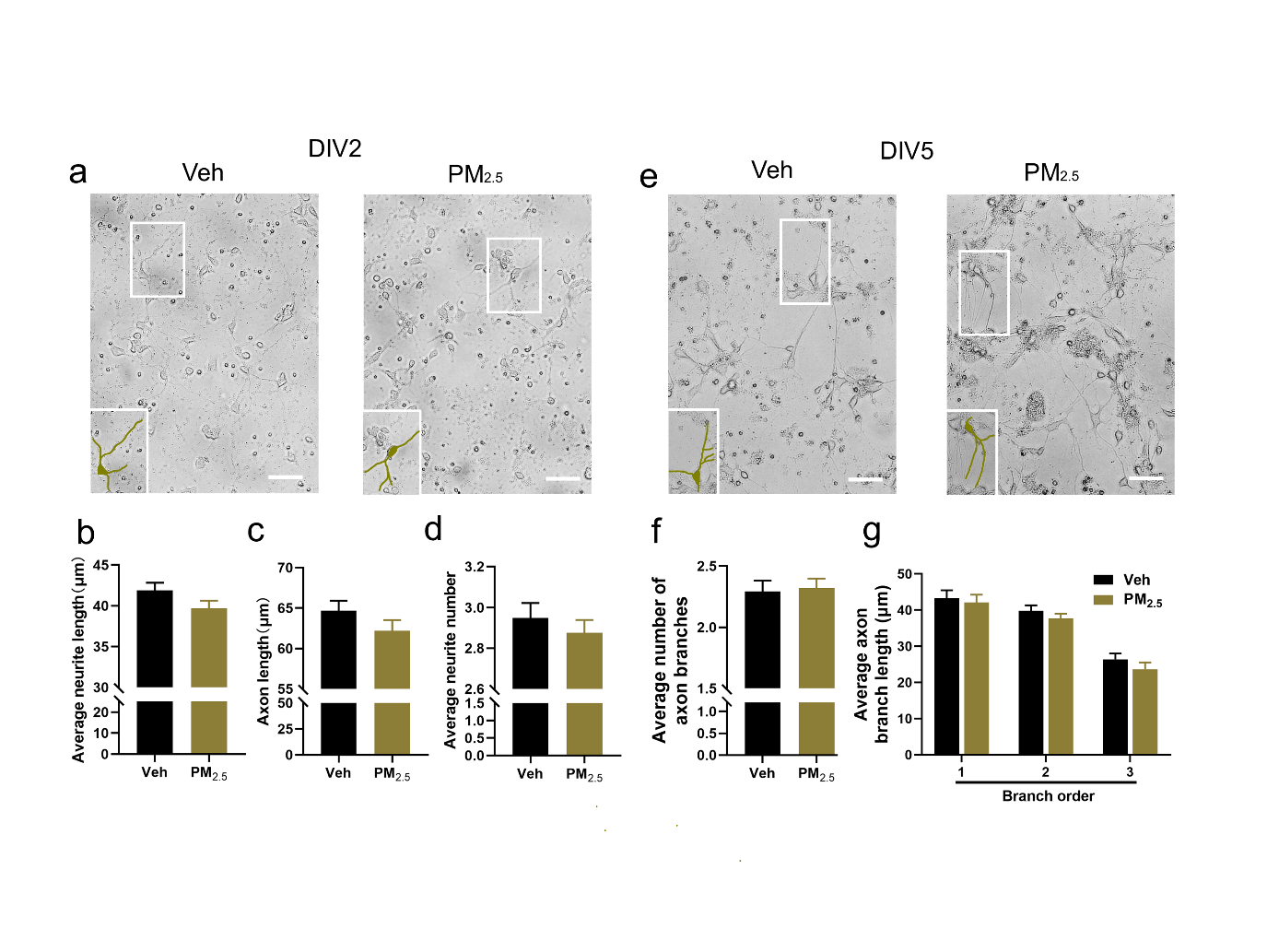
Fig. S4. Effects of prenatal PM_2.5_ exposure on morphological development of axons in female offspring. (a) Representative images of primary cultured cortical neurons on DIV2. Bar = 50 μm; (b) Average neurite length; (c) Axon length; and (d) Average neurite number (n = 97 (Veh) and 96 (PM_2.5_) neurons from 3 to 5 mice of different litters). (e) Representative images of primary cultured cortical neurons on DIV5. Bar = 50 μm; (f) Average number of axon branches; (g) Average axon branch length at different branches (n = 48 (Veh) and 50 (PM_2.5_) neurons from 3 to 5 mice of different litters).


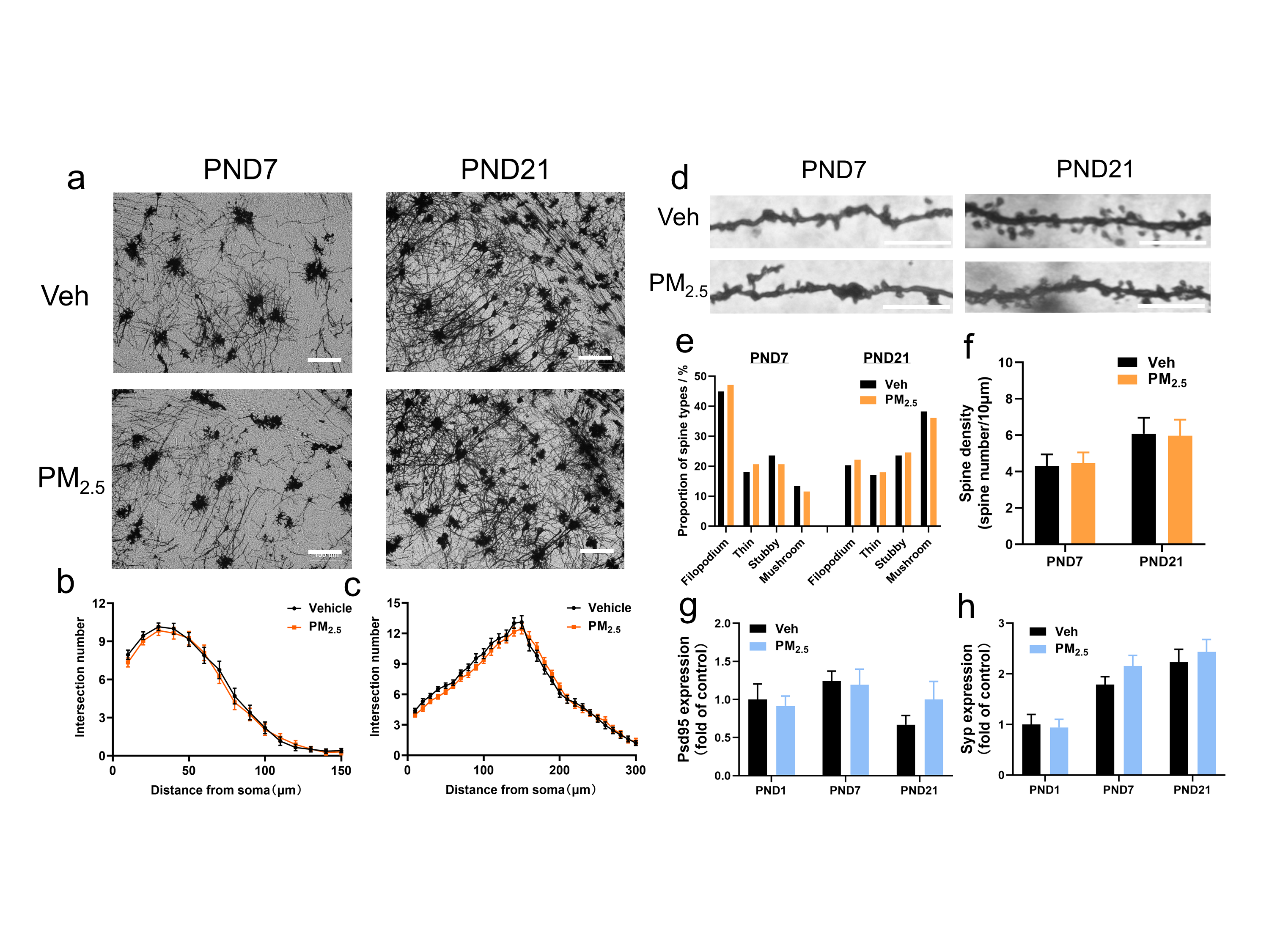
 Fig. S5. Effects of prenatal PM_2.5_ exposure on morphological development of dendrites and mRNA expression of synaptic markers in female offspring. (a) Representative images of Golgi-Cox-stained dendrites on PNDs 7 and 21. Bar = 200 μm; (b) Sholl analyses of dendritic complexity on PND 7 (n = 20 neurons from 3 mice of different litters); (c) Sholl analyses of dendritic complexity on PND 21 (n = 20 neurons from 3 mice of different litters). (d) Representative images of Golgi-Cox stained dendritic spines on PNDs 7 and 21. Bar = 10 μm; (e) The percentage of each spine morphology category (filopodium, thin, stubby, and mushroom) on PNDs 7 and 21 (n = 15 neurons from 3 mice of different litters); (f) Quantification of dendritic spine density calculated by the number of spines per 10-μm dendritic length (n = 20 neurons from 3 mice of different litters). (g) mRNA expression of Psd95 on PNDs 1, 7 and 21. (h) mRNA expression of Syp on PNDs 1, 7 and 21. 6 female offspring from different dams were used for each group.


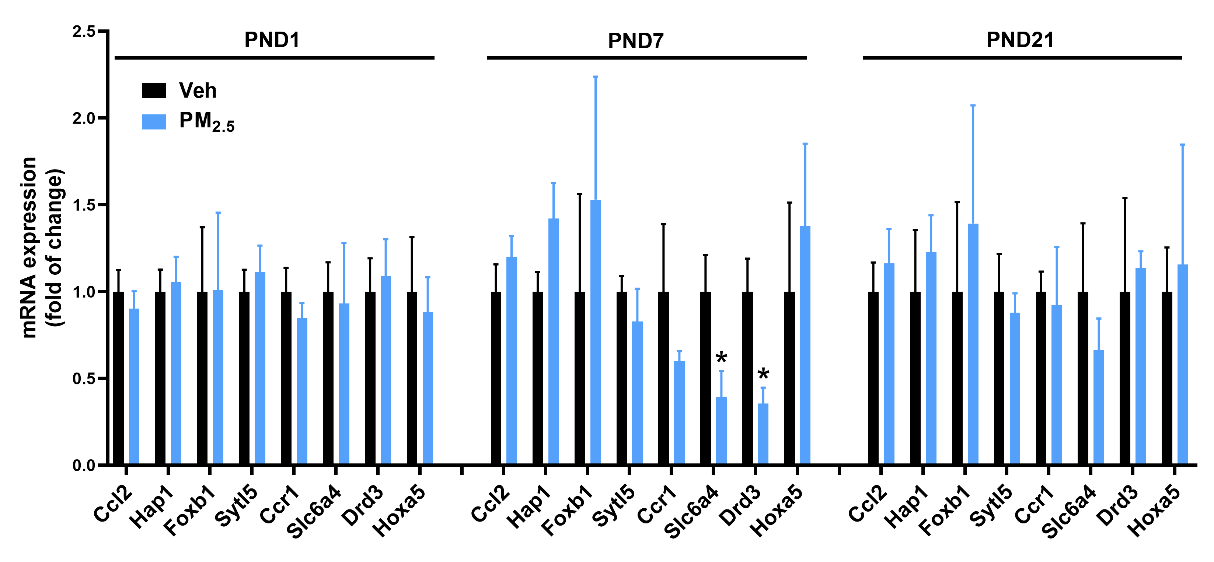
 Fig. S6. Effects of prenatal PM_2.5_ exposure on the expression of genes related to neuronal morphological development in female offspring. n = 4-6 from 4-6 litters. *p < 0. 05 for comparisons between the PM_2.5_ exposure group and the vehicle group.

Table S1. The contents of elements and polycyclic aromatic hydrocarbons in PM_2.5_ samples.

| Elements (ng/m^3^) | | Polycyclic aromatic hydrocarbon (PAHs) (ng/m^3^) | |
| --- | --- | --- | --- |
| Ca | 1397.85 | Benzo(b)fluoranthene (BbFA) | 16.70 |
| Fe | 1197.20 | Fluoranthene (FA) | 14.58 |
| K | 1066.67 | Chrysene (CHR) | 12.20 |
| Al | 735.48 | Benzo(a)anthracene (BaA) | 11.66 |
| Zn | 323.87 | Pyrene (PY) | 11.32 |
| Cu | 194.62 | Benzo(a)pyrene (BaP) | 9.76 |
| Pb | 97.94 | Indeno(1,2,3-cd)pyrene (IP) | 8.92 |
| Mn | 93.76 | Benzo(g,h,i)perylene (BghiP) | 8.59 |
| Mg | 43.01 | Phenanthrene (PHE) | 5.66 |
| W | 27.31 | Benzo(k)gluoranthene (BkFA) | 4.07 |
| Bi | 15.85 | Dibebz(a.h)anthracene (DBahA) | 1.71 |
| Cr | 11.83 | Fluorene (FL) | 0.54 |
| As | 5.59 | Anthracene (AN) | 0.49 |
| Sn | 4.84 | Naphthalene (NA) | 0.42 |
| Ni | 4.30 | Acenaphthylene (ACL) | 0.26 |
| Rb | 3.87 |  |  |
| Mo | 3.87 |  |  |
| Li | 2.56 |  |  |
| Sb | 1.42 |  |  |
| V | 1.38 |  |  |
| Ce | 1.10 |  |  |
| Tl | 1.07 |  |  |
| Co | 0.75 |  |  |
| Cd | 0.62 |  |  |
| La | 0.54 |  |  |
| Cs | 0.51 |  |  |
| Y | 0.28 |  |  |
| Th | 0.16 |  |  |
| U | 0.16 |  |  |
| Sm | 0.06 |  |  |
| Be | 0.04 |  |  |

Table S2. The top 30 downregulated DEGs associated with the development of axons, dendrites and synapses in mRNA-seq.

| **PND1** | **Gene ID** | **Gene Name** | **Fold change** | ***p*-value** |
| --- | --- | --- | --- | --- |
| 1 | ENSMUSG00000034384 | BARHL2 | 0.659915917 | 0.011557896 |
| 2 | ENSMUSG00000027858 | TSPAN2 | 3.226331396 | 4.07E-05 |
| 3 | ENSMUSG00000031688 | POU4F2 | 0.045882388 | 0.006950685 |
| 4 | ENSMUSG00000035385 | CCL2 | 0.264518976 | 0.04615337 |
| 5 | ENSMUSG00000021609 | SLC6A3 | 0.211299061 | 0.000608288 |
| **PND7** | **Gene ID** | **Gene Name** | **Fold change** | ***p*-value** |
| 1 | ENSMUSG00000059246 | FOXB1 | 0.088965226 | 0.020556403 |
| 2 | ENSMUSG00000027833 | SHOX2 | 0.062286771 | 0.020527886 |
| 3 | ENSMUSG00000026826 | NR4A2 | 1.981405625 | 0.008575727 |
| 4 | ENSMUSG00000029193 | CCKAR | 0 | 0.012404853 |
| 5 | ENSMUSG00000034486 | GBX2 | 0 | 0.000460318 |
| 6 | ENSMUSG00000029304 | SPP1 | 0.388387442 | 2.76E-09 |
| 7 | ENSMUSG00000027858 | TSPAN2 | 0.282639718 | 1.88E-05 |
| 8 | ENSMUSG00000032259 | DRD2 | 0.571407604 | 0.042906686 |
| 9 | ENSMUSG00000037446 | TULP1 | 0.170128577 | 0.007005029 |
| 10 | ENSMUSG00000067768 | XLR4B | 0.289111751 | 0.010444708 |
| 11 | ENSMUSG00000020838 | SLC6A4 | 0.084704389 | 2.61E-06 |
| **PND21** | **Gene ID** | **Gene Name** | **Fold change** | ***p*-value** |
| 1 | ENSMUSG00000034384 | BARHL2 | 0 | 0.012429116 |
| 2 | ENSMUSG00000027833 | SHOX2 | 0.112057232 | 0.047735144 |
| 3 | ENSMUSG00000042258 | ISL1 | 0.038530347 | 0.001736538 |
| 4 | ENSMUSG00000037660 | GDF7 | 0 | 0.000820382 |
| 5 | ENSMUSG00000026826 | NR4A2 | 0.451365475 | 0.002952309 |
| 6 | ENSMUSG00000029193 | CCKAR | 0 | 0.027597638 |
| 7 | ENSMUSG00000029304 | SPP1 | 0.636469898 | 0.032483647 |
| 8 | ENSMUSG00000032259 | DRD2 | 0.275349315 | 0.003170057 |
| 9 | ENSMUSG00000029697 | FEZF1 | 0 | 0.023865559 |
| 10 | ENSMUSG00000019230 | LHX9 | 0.142180748 | 0.001173496 |
| 11 | ENSMUSG00000006930 | HAP1 | 0.467463495 | 0.007215805 |
| 12 | ENSMUSG00000020178 | ADORA2A | 0.374895159 | 0.030406023 |
| 13 | ENSMUSG00000057132 | RPGRIP1 | 0.468291429 | 0.03943512 |
| 14 | ENSMUSG00000059854 | HYDIN | 0.411006709 | 0.023048882 |
| 15 | ENSMUSG00000025804 | CCR1 | 0.365351443 | 0.015799254 |
| 16 | ENSMUSG00000020090 | NPFFR1 | 0.373193589 | 0.035746713 |
| 17 | ENSMUSG00000028004 | NPY2R | 0.477191007 | 0.030156183 |
| 18 | ENSMUSG00000054453 | SYTL5 | 0.491915363 | 0.026983642 |
| 19 | ENSMUSG00000026322 | HTR4 | 0.456075389 | 0.020625929 |
| 20 | ENSMUSG00000062372 | OTOF | 0.492957891 | 0.001081272 |
| 21 | ENSMUSG00000022705 | DRD3 | 0 | 0.029982348 |

Abbreviations: DEGs, differentially expressed genes; PND, postnatal day.

Fold change: the fold change of gene expression in prenatal PM_2.5_ exposed group compared to that of vehicle group. Cortical tissue collected from pups of prenatally exposed to sterile saline or PM_2.5_ suspension was used for mRNA-sequence analyses, n=3 (from 3 litters) for each time point time.

Table S3. Correlation analyses between hub genes and their TFs in the cortex of male offspring on PNDs 1, 7 and 21 following prenatal PM_2.5_ exposure.

| hub gene | CCL2 | | | | | | |
| --- | --- | --- | --- | --- | --- | --- | --- |
| TFs | C/EBPbeta [T00017] | myogenin [T00528] | c-Fos [T00122] | HOXA5 [T00377] | JunD [T00437] | c-Jun [T00131] | YY1 [T00865] |
|  | DEC2 [T05845] | Pax-5 [T01201] | NF-1 [T00537] | HES-1 [T01649] | USF-1 [T00877] |  |  |
| hub gene | NGF | | | | | | |
| TFs | c-Fos [T00122] | NF-AT4 [T01949] | C/EBPbeta [T00017] | GATA-2 [T01302] | HES-1 [T01649] | YY1 [T00865] | HOXA5 [T00377] |
|  | GR  [T00335] | c-Jun [T00131] | JunD [T00437] | C/EBPalpha [T00104] | MyoD [T00526] | f(alpha)-f(epsilon) [T00287] | AP-1 [T00032] |
|  | NF-1 [T00537] |  |  |  |  |  |  |
| hub gene | NR4A2 | | | | | | |
| TFs | C/EBPbeta [T00017] | c-Fos [T00122] | HOXA5 [T00377] | C/EBPalpha [T00104] | HES-1 [T01649] | JunD [T00437] | YY1 [T00865] |
| hub gene | SHH | | | | | | |
| TFs | C/EBPbeta [T00017] | C/EBPalpha [T00104] | c-Fos [T00122] | c-Jun [T00131] | myogenin [T00528] | NF-1 [T00537] | HES-1 [T01649] |
|  | HOXA5 [T00377] | YY1 [T00865] |  |  |  |  |  |
| hub gene | SLIT3 | | | | | | |
| TFs | c-Fos [T00122] | myogenin [T00528] | C/EBPbeta [T00017] | USF-1 [T00877] | HOXA5 [T00377] | YY1 [T00865] | f(alpha)-f(epsilon) [T00287] |
|  | NF-1 [T00537] | C/EBPalpha [T00104] | JunD [T00437] | c-Jun [T00131] |  |  |  |
| hub gene | DRD2 | | | | | | |
| TFs | C/EBPbeta [T00017] | c-Fos [T00122] | HOXA5 [T00377] | YY1 [T00865] | C/EBPalpha [T00104] | NF-AT1 [T01944] | f(alpha)-f(epsilon) [T00287] |
|  | Pax-5 [T01201] | myogenin [T00528] |  |  |  |  |  |
| hub gene | CALB1 | | | | | | |
| TFs | C/EBPbeta [T00017] | HOXA5 [T00377] | c-Fos [T00122] | GR  [T00335] | c-Jun [T00131] | YY1 [T00865] | C/EBPalpha [T00104] |
|  | f(alpha)-f(epsilon) [T00287] | JunD [T00437] | MyoD [T00526] |  |  |  |  |
| hub gene | SLC17A8 | | | | | | |
| TFs | c-Fos [T00122] | C/EBPbeta [T00017] | C/EBPalpha [T00104] | HOXA5 [T00377] | YY1 [T00865] | c-Jun [T00131] | myogenin [T00528] |
|  | f(alpha)-f(epsilon) [T00287] |  |  |  |  |  |  |
| hub gene | NPY2R | | | | | | |
| TFs | C/EBPbeta [T00017] | c-Fos [T00122] | myogenin [T00528] | YY1 [T00865] | c-Jun [T00131] | HOXA5 [T00377] | C/EBPalpha [T00104] |
| hub gene | MAPT | | | | | | |
| TFs | NF-1 [T00537] | C/EBPbeta [T00017] | c-Fos [T00122] | YY1 [T00865] | C/EBPalpha [T00104] | myogenin [T00528] | HOXA5 [T00377] |
|  | TCF-1(P) [T01109] |  |  |  |  |  |  |
| hub gene | CNTN2 | | | | | | |
| TFs | C/EBPbeta [T00017] | HOXA5 [T00377] | JunD [T00437] | HES-1 [T01649] | c-Fos [T00122] | YY1 [T00865] | C/EBPalpha [T00104] |
| hub gene | NOS1 | | | | | | |
| TFs | C/EBPbeta [T00017] | myogenin [T00528] | HOXA5 [T00377] | c-Fos [T00122] | AP-1 [T00032] | c-Jun [T00131] | GATA-2 [T01302] |
|  | USF-1 [T00877] | YY1 [T00865] | C/EBPalpha [T00104] |  |  |  |  |
| hub gene | ETV1 | | | | | | |
| TFs | C/EBPbeta [T00017] | c-Fos [T00122] | NF-1 [T00537] | c-Jun [T00131] | C/EBPalpha [T00104] | PU.1 [T00702] | myogenin [T00528] |
|  | HOXA5 [T00377] | YY1 [T00865] |  |  |  |  |  |
| hub gene | ESR1 | | | | | | |
| TFs | c-Fos [T00122] | C/EBPbeta [T00017] | C/EBPalpha [T00104] | HOXA5 [T00377] | AP-1 [T00032] | GR [T00335] | HES-1 [T01649] |
|  | myogenin [T00528] | JunD [T00437] | c-Jun [T00131] | NF-kappaB [T00588] | NF-1 [T00537] | YY1 [T00865] |  |
| hub gene | ADCYAP1 | | | | | | |
| TFs | C/EBPbeta [T00017] | c-Fos [T00122] | HOXA5 [T00377] | NF-1 [T00537] | NF-AT4 [T01949] | c-Jun [T00131] | JunD [T00437] |
|  | YY1 [T00865] | AP-1 [T00032] | myogenin [T00528] | C/EBPalpha [T00104] | NF-kappaB [T00588] | HES-1 [T01649] |  |
| hub gene | ISL1 | | | | | | |
| TFs | C/EBPbeta [T00017] | c-Fos [T00122] | JunD [T00437] | HOXA5 [T00377] | NF-1 [T00537] | GR [T00335] | c-Jun [T00131] |
|  | myogenin [T00528] | NF-kappaB [T00588] | f(alpha)-f(epsilon) [T00287] |  |  |  |  |

Table S4. Prediction of binding sites between Hoxa5 and hub genes in the cortex of male offspring on PNDs 1, 7 and 21 following prenatal PM_2.5_ exposure.

|  | Score | Sequence ID | Start | End | Predicted sequence |
| --- | --- | --- | --- | --- | --- |
| **CCL2** | 9.440825 | NC_000077.7:81924403-81926503 | 1725 | 1732 | CAGTAATT |
|  | 9.177603 | NC_000077.7:81924403-81926503 | 1317 | 1324 | CTCTAATT |
|  | 8.92601 | NC_000077.7:81924403-81926503 | 417 | 424 | CAGAAATT |
|  | 7.732699 | NC_000077.7:81924403-81926503 | 926 | 933 | CTGAAATG |
|  | 7.117391 | NC_000077.7:81924403-81926503 | 144 | 151 | CAGTAGTT |
| **SLC17A8** | 8.92601 | NC_000076.7:c89459111-89457011 | 164 | 171 | CAGAAATT |
|  | 8.826468 | NC_000076.7:c89459111-89457011 | 1480 | 1487 | CACAAATG |
|  | 8.247515 | NC_000076.7:c89459111-89457011 | 1173 | 1180 | CTGTAATG |
|  | 6.854168 | NC_000076.7:c89459111-89457011 | 147 | 154 | CATTTATT |
|  | 6.186548 | NC_000076.7:c89459111-89457011 | 599 | 606 | CGGGAATG |
| **SHH** | 7.216953 | NC_000071.7:c28674099-28671999 | 1030 | 1037 | AGCTAATT |
|  | 6.854168 | NC_000071.7:c28674099-28671999 | 369 | 376 | CTCTTATT |
|  | 6.339353 | NC_000071.7:c28674099-28671999 | 1624 | 1631 | CATAAGTT |
|  | 6.338598 | NC_000071.7:c28674099-28671999 | 429 | 436 | CATAAATC |
|  | 6.113804 | NC_000071.7:c28674099-28671999 | 2052 | 2059 | CAGCAATT |
| **SLIT3** | 9.856098 | NC_000077.7:35010283-35012383 | 1173 | 1180 | CACTAATT |
|  | 7.984292 | NC_000077.7:35010283-35012383 | 92 | 99 | CTTTAATG |
|  | 6.854168 | NC_000077.7:35010283-35012383 | 1404 | 1411 | CATTTATT |
|  | 6.853414 | NC_000077.7:35010283-35012383 | 1124 | 1131 | CTCTAATC |
|  | 6.851147 | NC_000077.7:35010283-35012383 | 1177 | 1184 | CCCTAATT |
| **NGF** | 7.732699 | NC_000069.7:102375235-102377335 | 568 | 575 | CTGAAATG |
|  | 6.853414 | NC_000069.7:102375235-102377335 | 332 | 339 | CATTAATC |
|  | 6.702138 | NC_000069.7:102375235-102377335 | 157 | 164 | AAGAAATT |
|  | 6.538457 | NC_000069.7:102375235-102377335 | 1410 | 1417 | AGTTAATT |
|  | 6.503033 | NC_000069.7:102375235-102377335 | 178 | 185 | CACAAGTG |
| **CALB1** | 8.92601 | NC_000070.7:15879264-15881364 | 1535 | 1542 | CGCTAATG |
|  | 8.826468 | NC_000070.7:15879264-15881364 | 950 | 957 | CACAAATG |
|  | 8.662787 | NC_000070.7:15879264-15881364 | 1210 | 1217 | CTCTAATG |
|  | 7.984292 | NC_000070.7:15879264-15881364 | 1393 | 1400 | CTTAAATT |
|  | 7.117411 | NC_000070.7:15879264-15881364 | 1355 | 1362 | AACTAATG |
| **CNTN2** | 9.341283 | NC_000067.7:c132472989-132470889 | 1026 | 1033 | CACAAATT |
|  | 8.662787 | NC_000067.7:c132472989-132470889 | 1749 | 1756 | CATAAATT |
|  | 8.247515 | NC_000067.7:c132472989-132470889 | 2082 | 2089 | CTGAAATT |
|  | 7.117411 | NC_000067.7:c132472989-132470889 | 644 | 651 | AACAAATT |
|  | 7.117411 | NC_000067.7:c132472989-132470889 | 1804 | 1811 | AACAAATT |
| **MAPT** | 9.341283 | NC_000077.7:104118235-104120335 | 1102 | 1109 | CACTAATG |
|  | 7.532663 | NC_000077.7:104118235-104120335 | 1482 | 1489 | CACTTATT |
|  | 7.469477 | NC_000077.7:104118235-104120335 | 1327 | 1334 | CTTAAATG |
|  | 7.117411 | NC_000077.7:104118235-104120335 | 1449 | 1456 | AACAAATT |
|  | 7.117391 | NC_000077.7:104118235-104120335 | 337 | 344 | CAGTTATT |
| **NOS1** | 7.531909 | NC_000071.7:118002904-118005004 | 292 | 299 | CACTAATC |
|  | 7.216953 | NC_000071.7:118002904-118005004 | 1004 | 1011 | AAGTAATT |
|  | 7.017848 | NC_000071.7:118002904-118005004 | 1253 | 1260 | CACTAGTG |
|  | 7.017848 | NC_000071.7:118002904-118005004 | 1253 | 1260 | CACTAGTG |
|  | 6.702117 | NC_000071.7:118002904-118005004 | 1209 | 1216 | CGGTAGTT |
| **ESR1** | 9.440825 | NC_000076.7:4559989-4562089 | 658 | 665 | CGCTAATT |
|  | 9.341283 | NC_000076.7:4559989-4562089 | 900 | 907 | CACTAATG |
|  | 9.177603 | NC_000076.7:4559989-4562089 | 1128 | 1135 | CTCTAATT |
|  | 8.826468 | NC_000076.7:4559989-4562089 | 739 | 746 | CACAAATG |
|  | 7.732699 | NC_000076.7:4559989-4562089 | 676 | 683 | CTGAAATG |
| **DRD2** | 8.499107 | NC_000075.7:49249633-49251733 | 576 | 583 | CTTTAATT |
|  | 7.469477 | NC_000075.7:49249633-49251733 | 435 | 442 | CTTAAATG |
|  | 7.017094 | NC_000075.7:49249633-49251733 | 663 | 670 | CACAAATC |
|  | 6.187323 | NC_000075.7:49249633-49251733 | 767 | 774 | AGCAAATG |
|  | 5.943927 | NC_000075.7:49249633-49251733 | 1818 | 1825 | CACTCATT |
| **NPY2R** | 9.177603 | NC_000069.7:c82458310-82456210 | 620 | 627 | CATTAATT |
|  | 7.469477 | NC_000069.7:c82458310-82456210 | 896 | 903 | CTTAAATG |
|  | 7.117411 | NC_000069.7:c82458310-82456210 | 1831 | 1838 | AACTAATG |
|  | 6.854168 | NC_000069.7:c82458310-82456210 | 1831 | 1838 | CATTAGTT |
|  | 6.853414 | NC_000069.7:c82458310-82456210 | 1605 | 1612 | CATGAATT |
| **ETV1** | 9.440825 | NC_000078.7:38827655-38829755 | 269 | 276 | CAGTAATT |
|  | 7.469477 | NC_000078.7:38827655-38829755 | 1488 | 1495 | CTTAAATG |
|  | 5.824537 | NC_000078.7:38827655-38829755 | 1602 | 1609 | CTCAAGTG |
|  | 5.429112 | NC_000078.7:38827655-38829755 | 416 | 423 | CACACATT |
|  | 5.348037 | NC_000078.7:38827655-38829755 | 1823 | 1830 | CAGTAAGT |
| **ISL1** | 7.117411 | NC_000079.7:c116448224-116446124 | 857 | 864 | AACAAATT |
|  | 7.117391 | NC_000079.7:c116448224-116446124 | 746 | 753 | CAGTTATT |
|  | 6.851147 | NC_000079.7:c116448224-116446124 | 792 | 799 | CAATAATT |
|  | 6.702138 | NC_000079.7:c116448224-116446124 | 788 | 795 | AAGAAATT |
|  | 6.538457 | NC_000079.7:c116448224-116446124 | 1193 | 1200 | AGTTAATT |
| **ADCYAP1** | 8.92601 | NC_000083.7:93503673-93505773 | 1560 | 1567 | CAGAAATT |
|  | 8.92601 | NC_000083.7:93503673-93505773 | 1564 | 1571 | CAGAAATT |
|  | 8.662787 | NC_000083.7:93503673-93505773 | 377 | 384 | CTCAAATT |
|  | 8.499107 | NC_000083.7:93503673-93505773 | 234 | 241 | CTTTAATT |
|  | 8.247515 | NC_000083.7:93503673-93505773 | 1578 | 1585 | CTGAAATT |
| **NR4A2** | 7.469477 | NC_000068.8:c57016018-57013918 | 1506 | 1513 | CTTAAATG |
|  | 7.117391 | NC_000068.8:c57016018-57013918 | 642 | 649 | CAGTTATT |
|  | 6.601821 | NC_000068.8:c57016018-57013918 | 818 | 825 | CAGAAATC |
|  | 6.438915 | NC_000068.8:c57016018-57013918 | 448 | 455 | AATAAATT |
|  | 6.43814 | NC_000068.8:c57016018-57013918 | 1164 | 1171 | CTGGAATT |

Table S5. Primers used in this study.

| Gene ID | Gene Name | Primer Sequences |
| --- | --- | --- |
| 13385 | Psd95 | Forward: 5′-TCCGGGAGGTGACCCATTC-3′  Reverse: 5′-TTTCCGGCGCATGACGTAG-3′ |
| 20977 | Syp | Forward: 5′-AGACATGGACGTGGTGAATCA-3′  Reverse: 5′-ACTCTCCGTCTTGTTGGCAC-3′ |
| 12425 | Cckar | Forward: 5′-GACAGCCTTCTTATGAATGGGAG-3′  Reverse: 5′-GCTGAGGTTGATCCAGGCAG-3′ |
| 104382 | Barhl2 | Forward: 5′-GCGGGTCGAGTTTTGGAATAG-3′  Reverse: 5′-GCTCCTAAAATCCGTTGTCCTC-3′ |
| 238057 | Gdf7 | Forward: 5′-GAGCTTCCTGTTCGACGTATC-3′  Reverse: 5′-CAGGCAGAACTTGCGGGAG-3′ |
| 73191 | Fesf1 | Forward: 5′-ACGCGACCACCAAAATGCTA-3′  Reverse: 5′-TCGACGAGTTGAGATGCAGAG-3′ |
| 13490 | Drd3 | Forward: 5′-TGGGGCAGAAAACTCCACTG-3′  Reverse: 5′-TACCAGACCGTTGCCAAAGAT-3′ |
| 16392 | Isl1 | Forward: 5′-TTTCCCTGTGTGTTGGTTGC-3′  Reverse: 5′-TGATTACACTCCGCACATTTCA-3′ |
| 18997 | Pou4f2 | Forward: 5′-TGGACATCGTCTCCCAGAGTA-3′  Reverse: 5′-GTGTTCATGGTGTGGTAAGTGG-3′ |
| 14472 | Gbx2 | Forward: 5′-GGGAGTAGTACCGCCTTCAG-3′  Reverse: 5′-CCGGTGTAGACGAAATGGC-3′ |
| 20429 | Shox2 | Forward: 5′-CAAAGACGATGCGAAAGGGAT-3′  Reverse: 5′-AGGGTAAAATTGGTCCGACTTC-3′ |
| 15567 | Slc6a4 | Forward: 5′-CTCCGCAGTTCCCAGTACAAG-3′  Reverse: 5′-CACGGCATAGCCAATGACAGA-3′ |
| 64290 | Foxb1 | Forward: 5′-CTTCAAGGTGCTCAAGTCAGAC-3′  Reverse: 5′-GTTCTCGATAGCAAAGGGATGC-3′ |
| 16876 | Lhx9 | Forward: 5′-ATGCTCTTCCACGGAATCTCC-3′  Reverse: 5′-CTACGGCCAGCAGATAGTACC-3′ |
| 22157 | Tulp1 | Forward: 5′-ATCCAAACCCCGTAAAGCTGG-3′  Reverse: 5′-CTTGGCGTAGACTGTCTGCG-3′ |
| 13162 | Slc6a3 | Forward: 5′-TTCATGGTTATTGCCGGGATG-3′  Reverse: 5′-TGTAGAAGAAGCCCACGTAGAA-3′ |
| 20296 | Ccl2 | Forward: 5′-TAAAAACCTGGATCGGAACCAAA-3′  Reverse: 5′-GCATTAGCTTCAGATTTACGGGT-3′ |
| 13489 | Drd2 | Forward: 5′-ACCTGTCCTGGTACGATGATG-3′  Reverse: 5′-GCATGGCATAGTAGTTGTAGTGG-3′ |
| 70747 | Tspan2 | Forward: 5′-TATCTGCTGCTCGGCTTCAAC-3′  Reverse: 5′-GTCCAAATGCAATAACGGCTG-3′ |
| 27083 | Xlr4b | Forward: 5′-GTTGACCACTTCTTGAAAGTCCA-3′  Reverse: 5′-CAGAGAGTTTTCCAGCCTGTTT-3′ |
| 12768 | Ccr1 | Forward: 5′-ACTGCTGTAAGAGCCTTTGGG-3′  Reverse: 5′-AGCACCAGAATCACTAGGACA-3′ |
| 237362 | Npffr1 | Forward: 5′-CCCTCGTGGACAACCTTATCA-3′  Reverse: 5′-CAGTGTGAAAACCGACGCA-3′ |
| 11540 | Adora2a | Forward: 5′-GGGCCGTGTGGATCAACAG-3′  Reverse: 5′-TGTCGATGGCAATAGCCAAGA-3′ |
| 20750 | Spp1 | Forward: 5′-ATCTCACCATTCGGATGAGTCT-3′  Reverse: 5′-TGTAGGGACGATTGGAGTGAAA-3′ |
| 244653 | Hydin | Forward: 5′-AAAATCCCAAACGTGCTCTTCT-3′  Reverse: 5′-GGGTGGTAAGACTTTGCTCTG-3′ |
| 18227 | Nr4a2 | Forward: 5′-GTGTTCAGGCGCAGTATGG-3′  Reverse: 5′-TGTATTCTCCCGAAGAGTGGTAA-3′ |
| 15562 | Htr4 | Forward: 5′-GATGCTAATGTGAGTTCCAACGA-3′  Reverse: 5′-CAGCAGGTTGCCCAAGATG-3′ |
| 15114 | Hap1 | Forward: 5′-ATCATTGGTGATTCGGACGCA-3′  Reverse: 5′-ATTAGGACACAACGCTTCCTG-3′ |
| 77945 | Rpgrip1 | Forward: 5′-GAGACACAGATTCGAGTCCTCT-3′  Reverse: 5′-GTAGGTCCGGTTGGGTAGTTG-3′ |
| 18167 | Npy2r | Forward: 5′-ACCGCCATCGTTGCATTGT-3′  Reverse: 5′-TCAGGGAGTATTCCCGGAAGA-3′ |
| 236643 | Sytl5 | Forward: 5′-GGAAGACAAGAGGATAAGGAAGC-3′  Reverse: 5′-TTGTGACAGTGAACGCAGACT-3′ |
| 83762 | Otof | Forward: 5′-CTGACACGGCATTCGTCTG-3′  Reverse: 5′-CCTGGGAGGCTGTAAAGGAA-3′ |
| 12608 | C/EBPbeta | Forward: 5′-TTATAAACCTCCCGCTCGGC  Reverse: 5′- TTCCATGGGTCTAAAGGCGG |
| 14281 | cFos | Forward: 5′-CGGGTTTCAACGCCGACTA  Reverse: 5′- TTGGCACTAGAGACGGACAGA |
| 15402 | Hoxa5 | Forward: 5′-CTCATTTTGCGGTCGCTATCC  Reverse: 5′- ATCCATGCCATTGTAGCCGTA |
| 14433 | Gapdh | Forward: 5′-CAAAATGGTGAAGGTCGGTGTG-3′  Reverse: 5′-TGATGTTAGTGGGGTCTCGCTC-3′ |

Text S1. Mating scheme of mice

After the adaptation period, female and male mice were mated (two females and one male per cage). The mice were paired for 3 days. The vaginal plug was examined in the daily morning, and if found, the event was counted as gestation day 0 (GD 0). On the fourth day morning, the males were removed, and a total of 75 pregnant female mice were randomly divided into a vehicle control group (37 mice) and a PM_2.5_ exposure group (38 mice), and they were individually housed until offspring weaning.

Text S2. The protocol for in vitro cytotoxicity assay

Primary neuron cells were seeded into 96-well plates with 100 μL complete neuronal media. The neurons were grown for 24 h at 37℃. Then 10 μL IC solution was added to the media to make the final concentration was 1, 3, 10, 100, 300 μg/mL, and 10 μL OC solution was added to the media to make the final concentration was 0.3, 1, 3, 5, 10, 30 μg/mL, respectively. After 24 h, 10 μL CCK-8 solution were added into each well, and the neurons were returned to the incubator for an additional 4 h. A Thermo Scientific Varioskan Flash reader (Thermo Fisher Scientific, USA) was used for detecting the absorbances at 450 nm. DMSO (0.1%) was used as the negative control in OC cytotoxicity experiments.

Excel table S1. Full list of neurodevelopment-related GO terms of biological process based on cortical DEGs in male offspring on PNDs 1, 7 and 21 following prenatal PM_2.5_ exposure.

Excel table S2. Full list of GO terms of biological process enriched by neuronal development-associated DEGs on PNDs 1, 7 and 21 in male offspring following prenatal PM_2.5_ exposure.
